# Supplementary material for: Ubiquitous MEIS transcription factors actuate lineage-specific transcription to establish cell fate
Source: EMBO J. 2025 Feb 28;44(8):2232–62. doi: 10.1038/s44318-025-00385-5 (PMC12000411; doi:10.1038/s44318-025-00385-5)
Supplement: Supplementary file 3 — Appendix [file 44318_2025_385_MOESM3_ESM.pdf]

Appendix

Appendix Figure S1.. p. 2

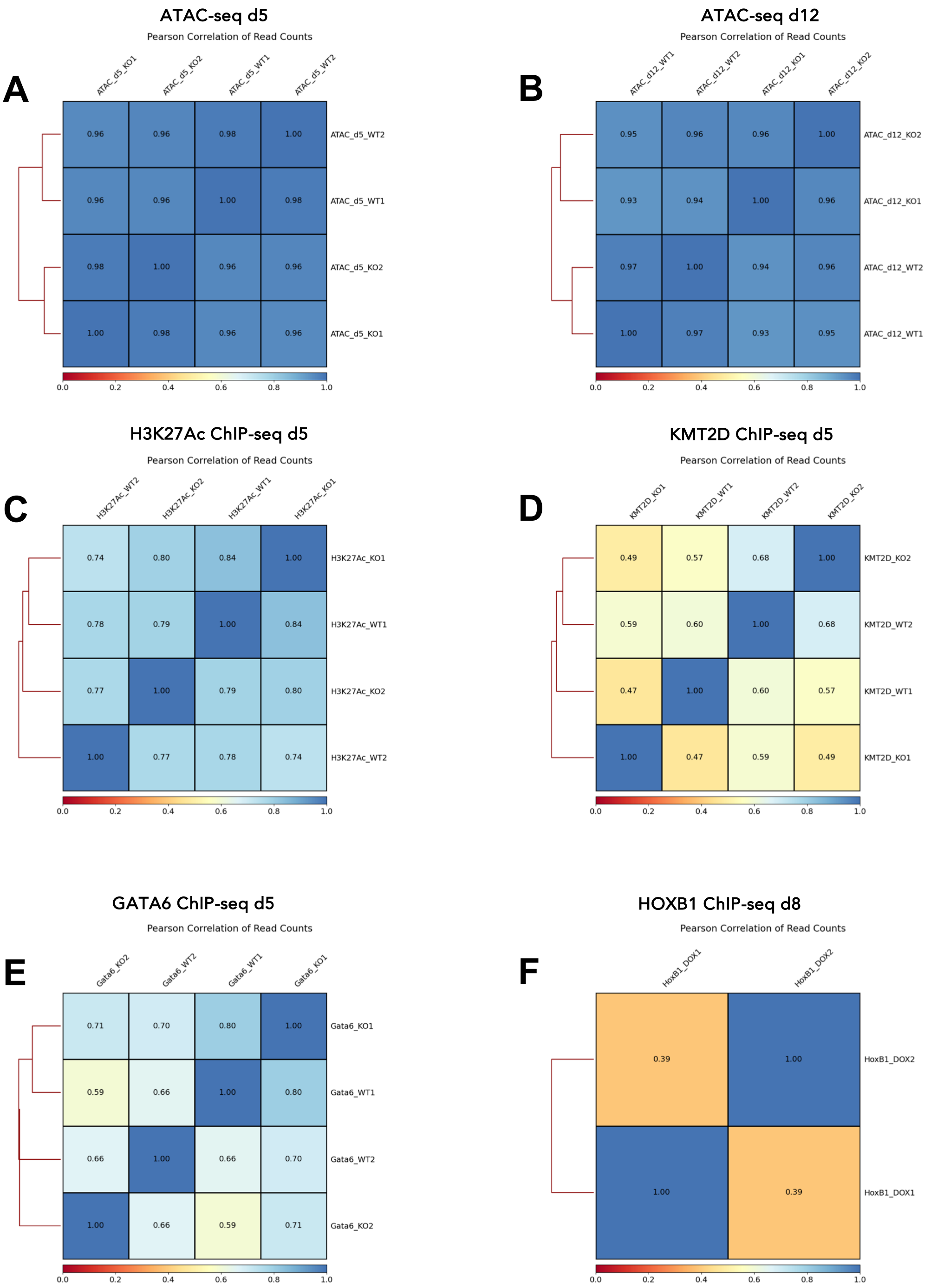

Appendix Figure S1. Pearson correlation of ATAC-seq (AB) and ChIP-seq replicated experiments (C-F). A. Correlation of four ATAC-seq replicates at d5: two WT and two MEIS KO. B. Correlation of four ATAC-seq replicates at d12: two WT and two MEIS KO. C-F. Correlation of ChIP-seq replicates performed at the same stage and using the same antibody. C. Four H3K27Ac ChIP-seq replicates at d5: two WT and two MEIS KO. D. Four KMT2D ChIP-seq replicates at d5: two WT and two MEIS KO. E. Four GATA6 ChIP-seq replicates at d5: two WT and two MEIS KO. F. Two HOXB1 ChIP-seq replicates at d5: WT. One HOXB1 replicate was lower quality. Unlike other ChIP-seq datasets used for quantitative comparisons and overlap analyses, HOXB1 ChIP-seq was primarily used to confirm HOX binding at predicted HOX-MEIS enhancers.
