# Supplementary figures and images for: Ubiquitous MEIS transcription factors actuate lineage-specific transcription to establish cell fate
Source: EMBO J. 2025 Feb 28;44(8):2232–62. doi: 10.1038/s44318-025-00385-5 (PMC12000411; doi:10.1038/s44318-025-00385-5)

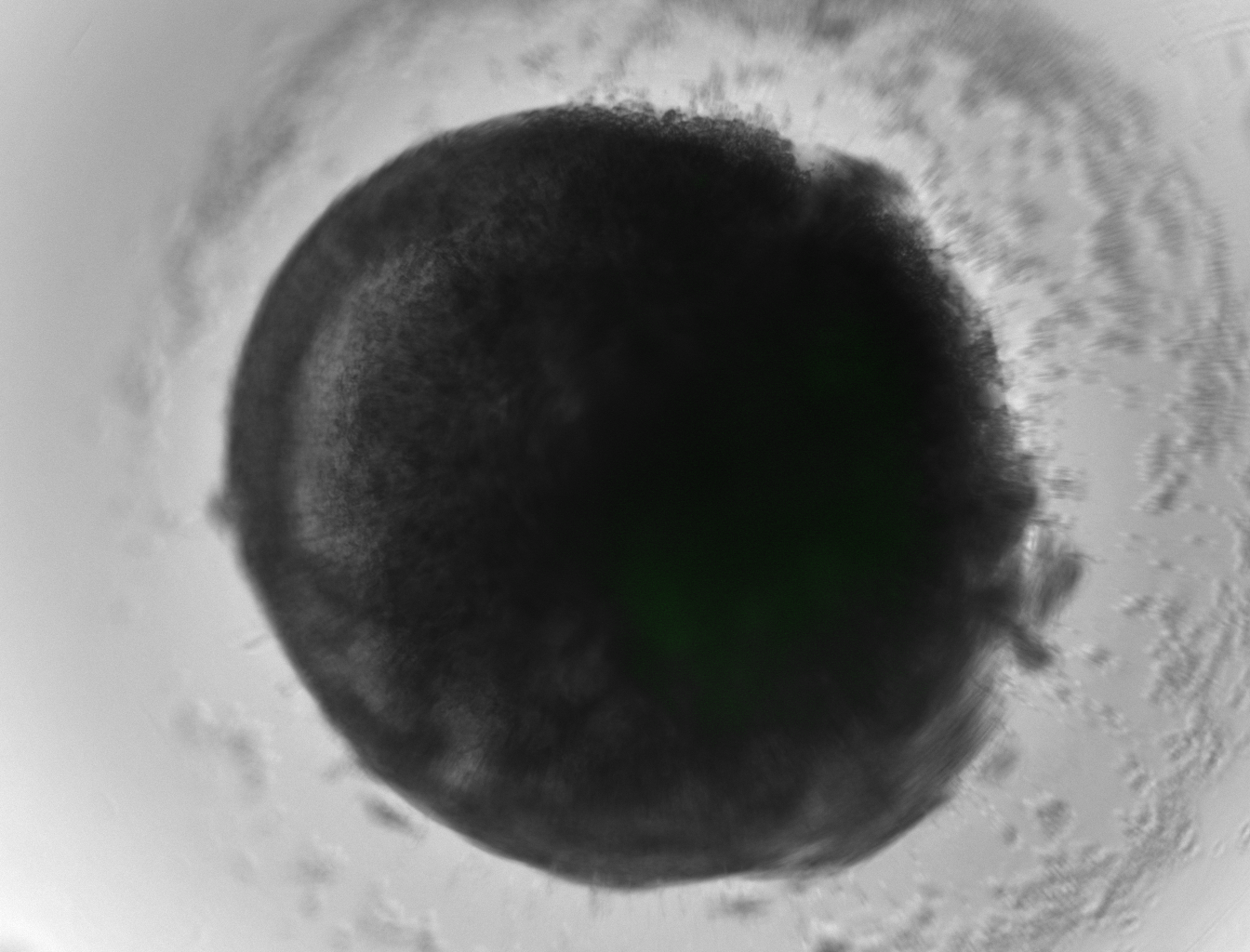

Supplement: Supplementary file 5 — Source data Fig. 2 [file 44318_2025_385_MOESM5_ESM.zip › Fig 2_Source_data/Source Figure 2A/2A_KO3.tif]

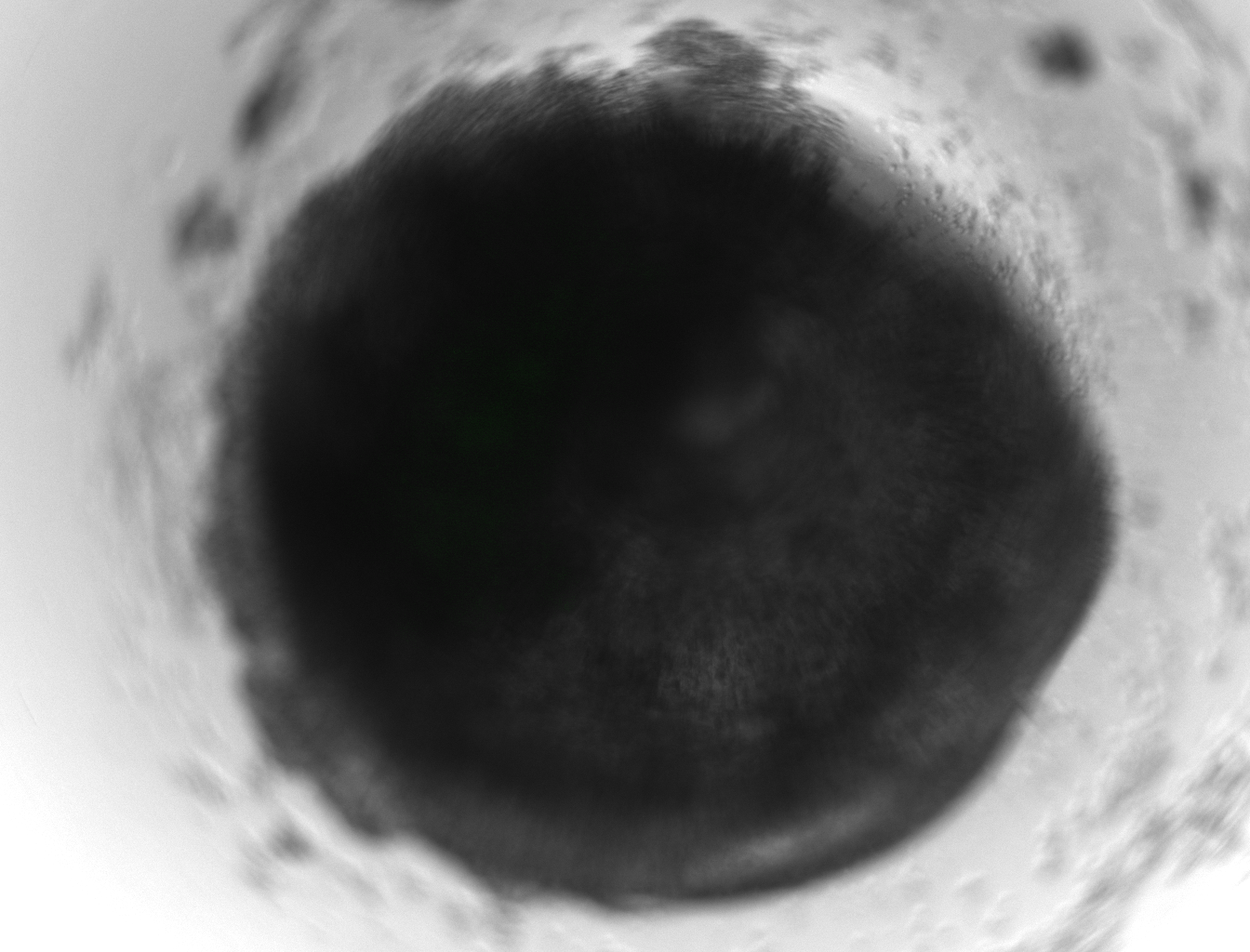

Supplement: Supplementary file 5 — Source data Fig. 2 [file 44318_2025_385_MOESM5_ESM.zip › Fig 2_Source_data/Source Figure 2A/2A_KO2.tif]

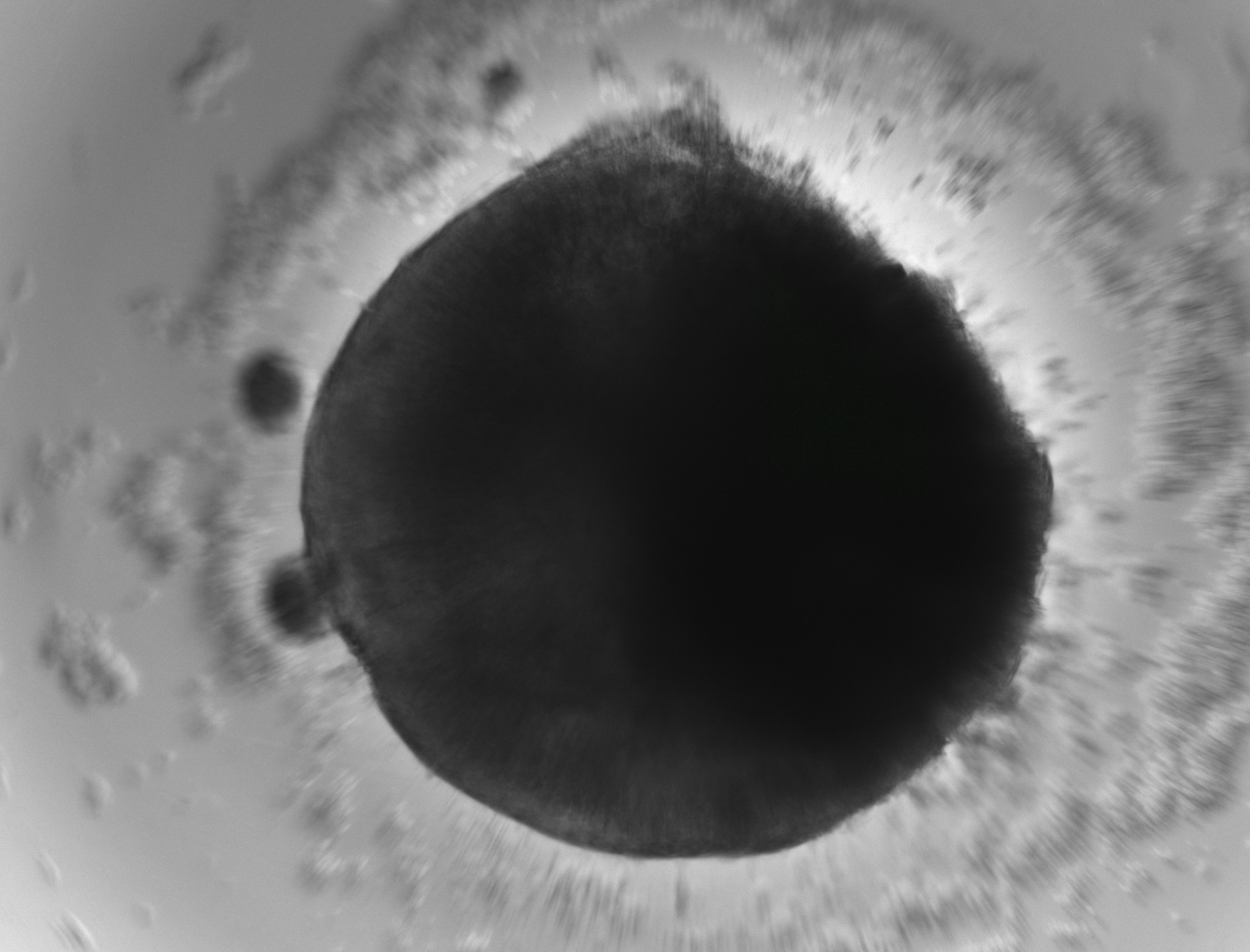

Supplement: Supplementary file 5 — Source data Fig. 2 [file 44318_2025_385_MOESM5_ESM.zip › Fig 2_Source_data/Source Figure 2A/2A_KO1.tif]

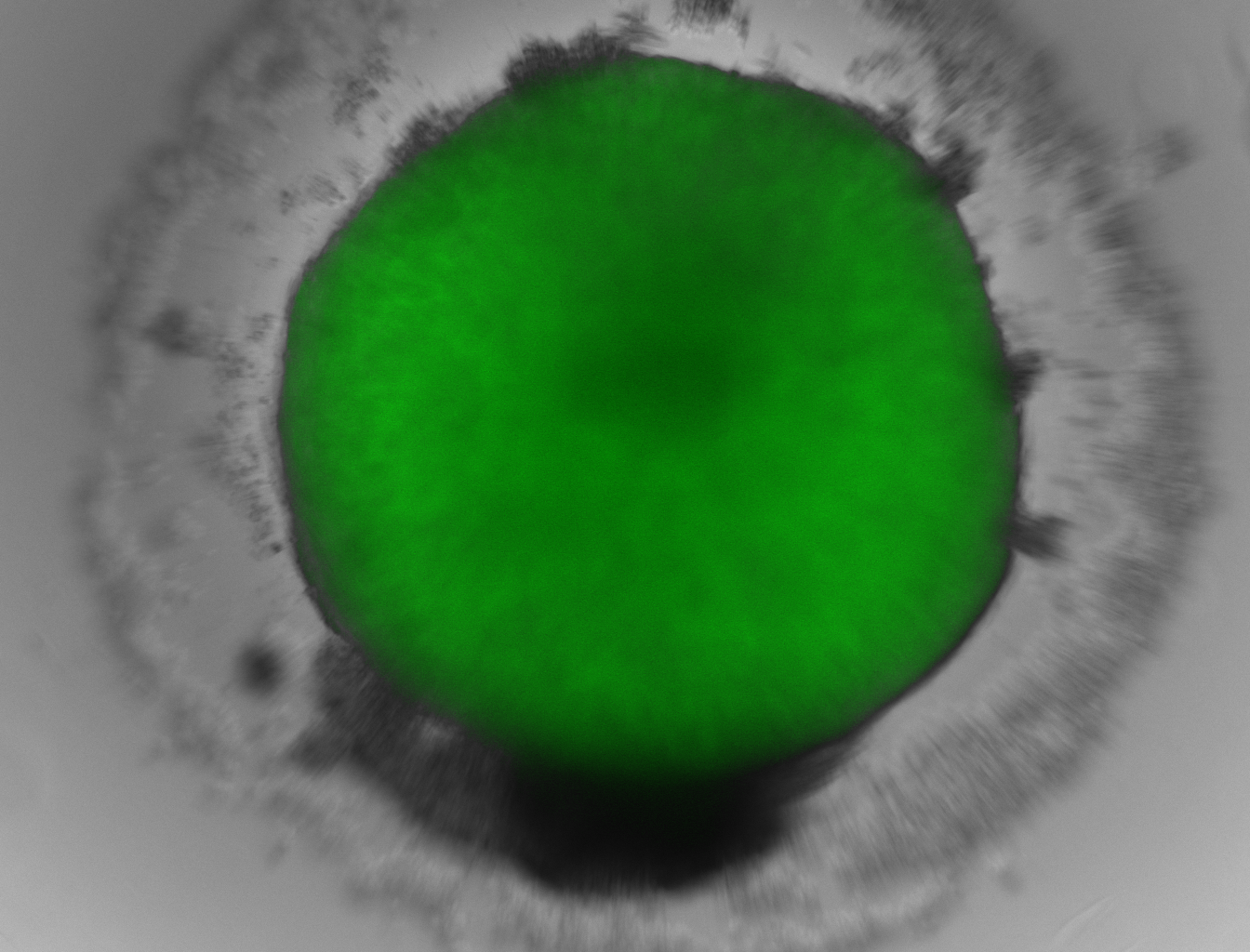

Supplement: Supplementary file 5 — Source data Fig. 2 [file 44318_2025_385_MOESM5_ESM.zip › Fig 2_Source_data/Source Figure 2A/2A_WT3.tif]

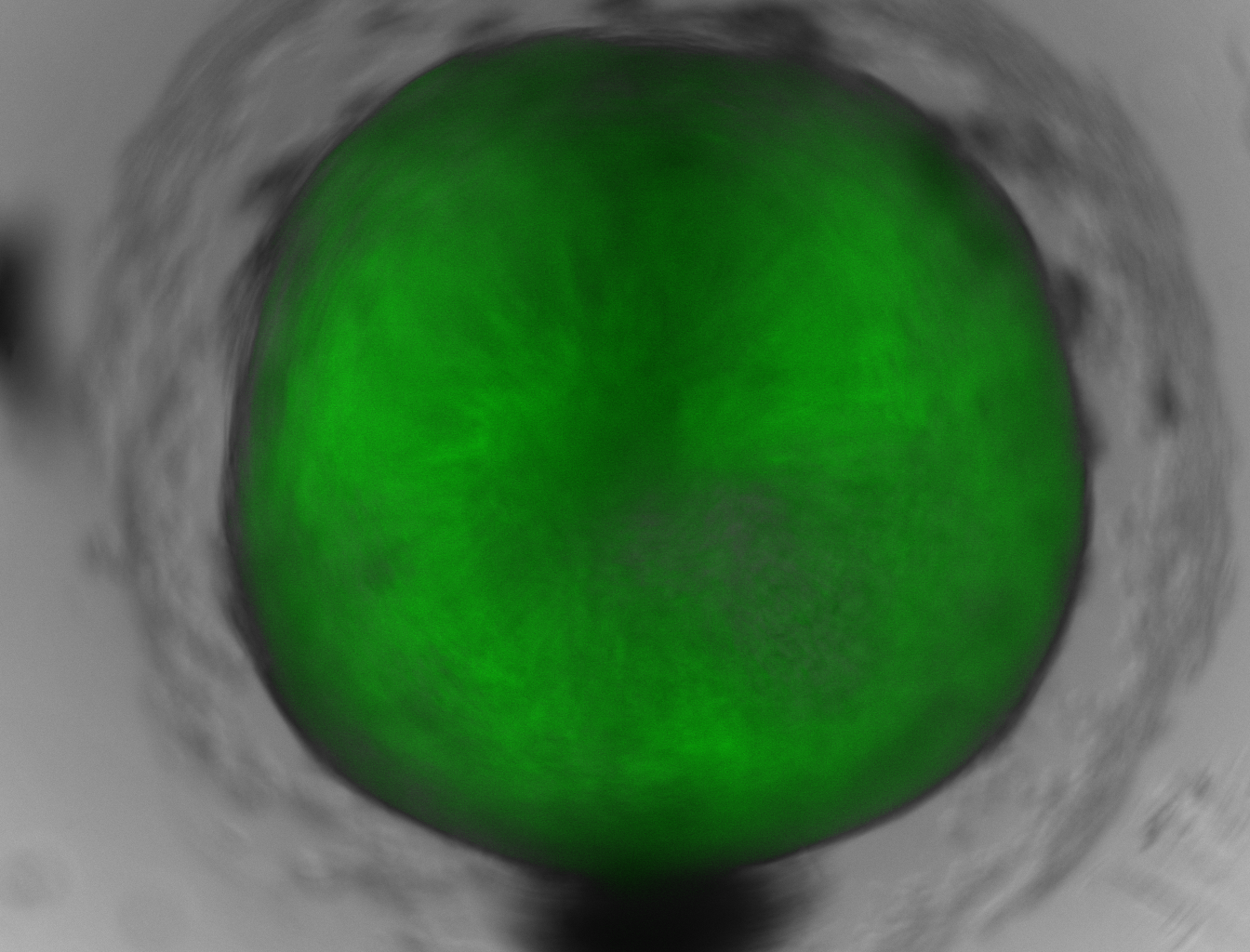

Supplement: Supplementary file 5 — Source data Fig. 2 [file 44318_2025_385_MOESM5_ESM.zip › Fig 2_Source_data/Source Figure 2A/2A_WT2.tif]

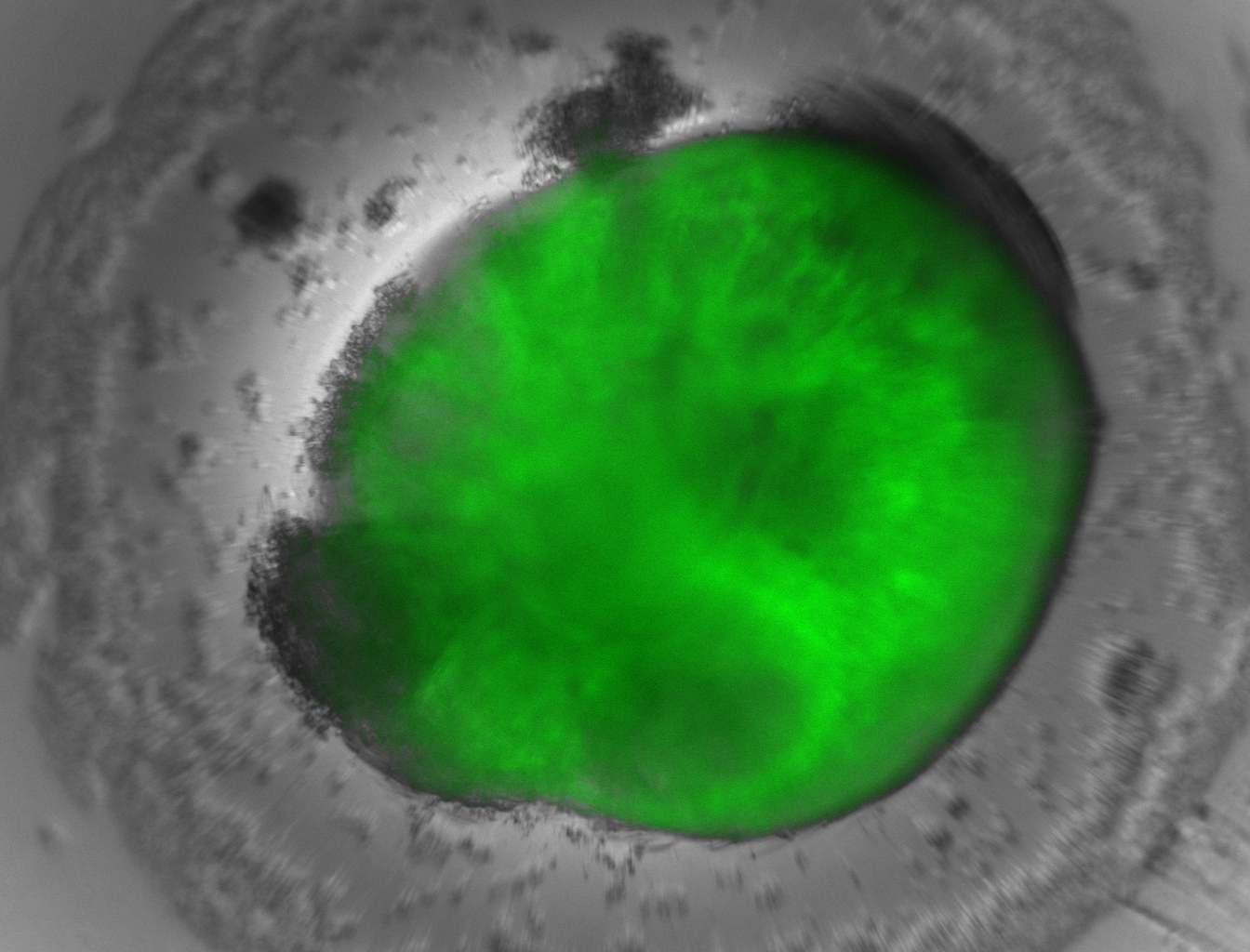

Supplement: Supplementary file 5 — Source data Fig. 2 [file 44318_2025_385_MOESM5_ESM.zip › Fig 2_Source_data/Source Figure 2A/2A_WT1.tif]

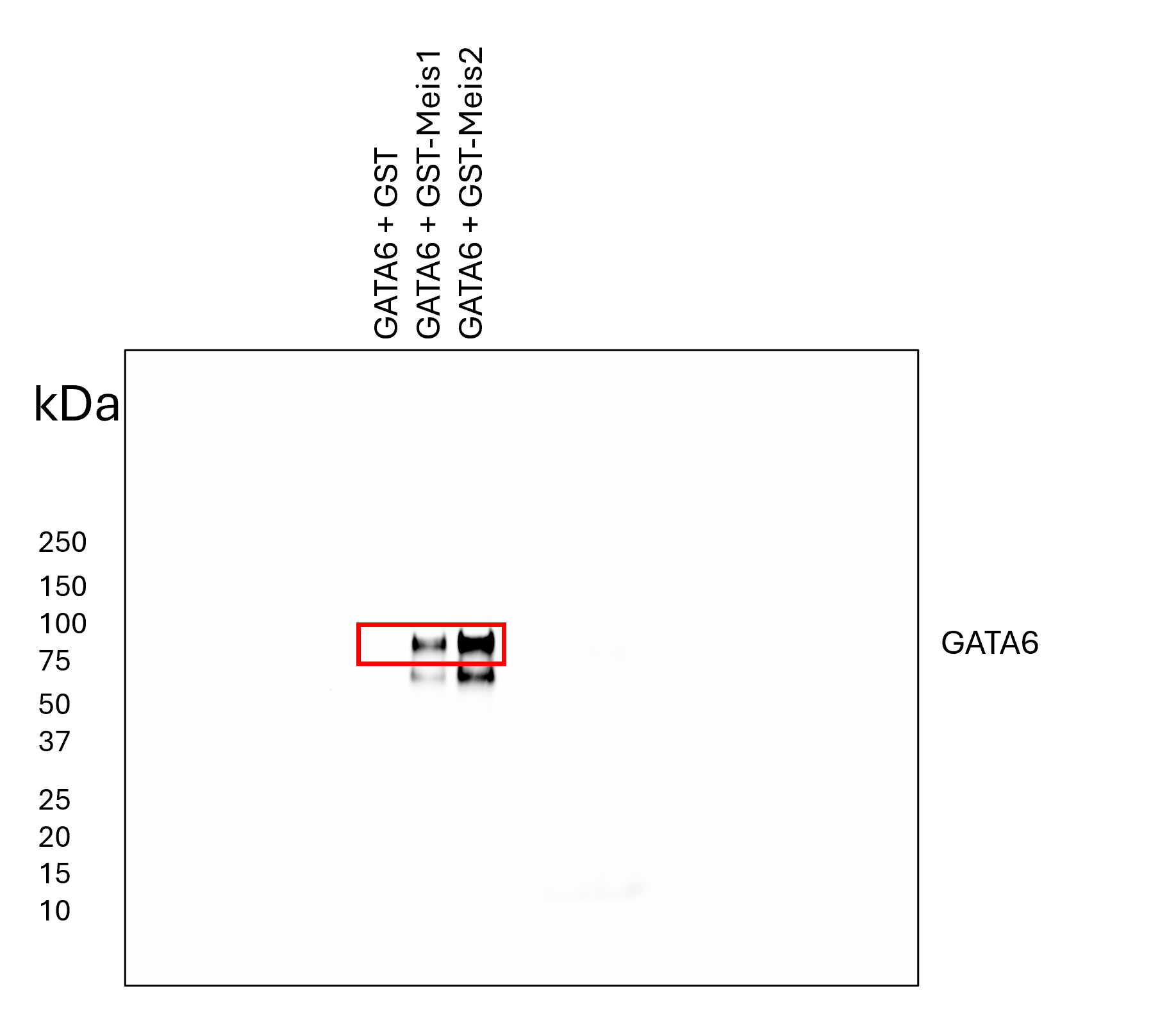

Supplement: Supplementary file 6 — Source data Fig. 4 [file 44318_2025_385_MOESM6_ESM.zip › Fig 4_Source_data/Source data Fig4H/western gata6 experiment gata6 CoP.tif]

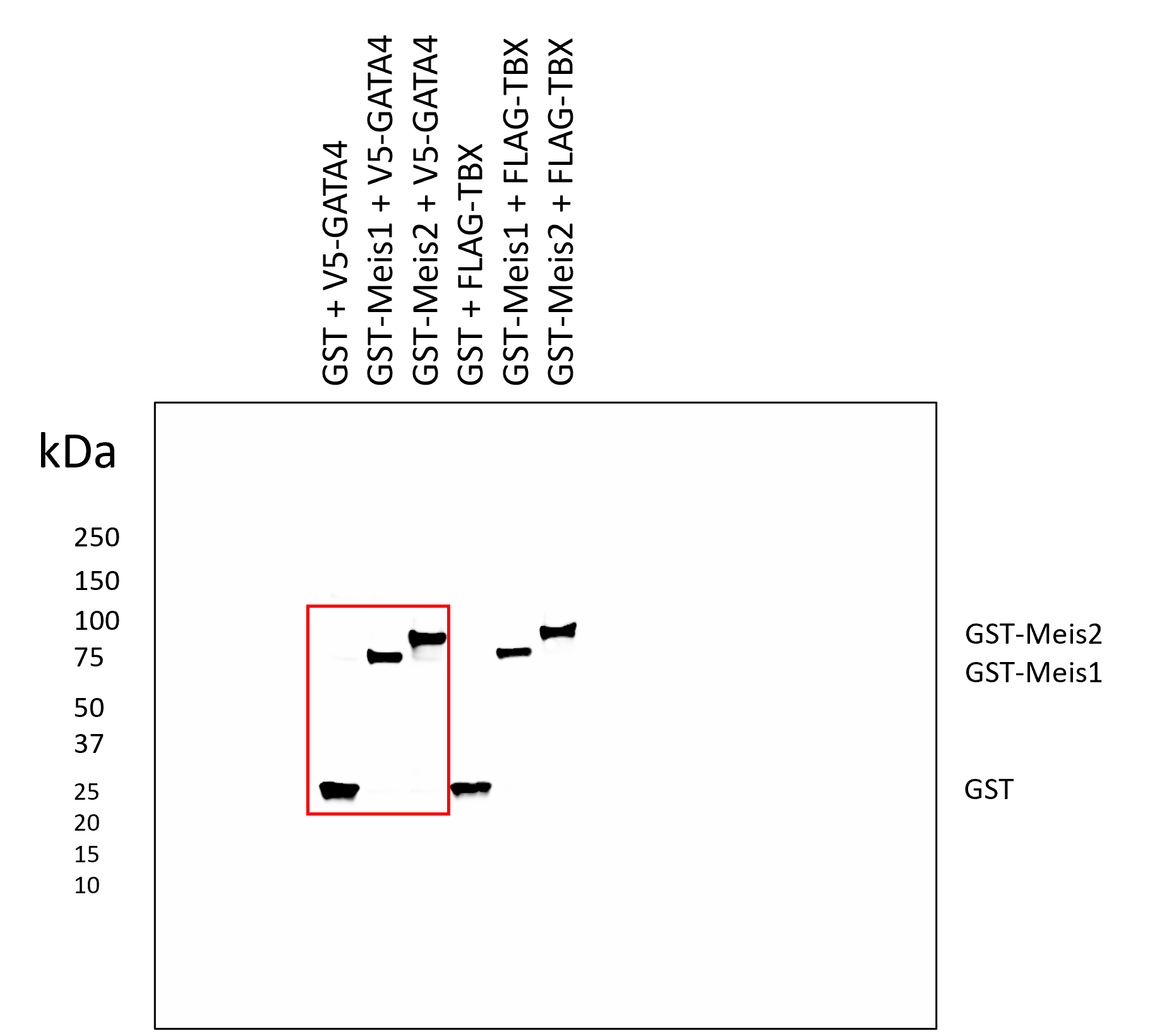

Supplement: Supplementary file 6 — Source data Fig. 4 [file 44318_2025_385_MOESM6_ESM.zip › Fig 4_Source_data/Source data Fig4H/western gata4 experiment gst.tif]

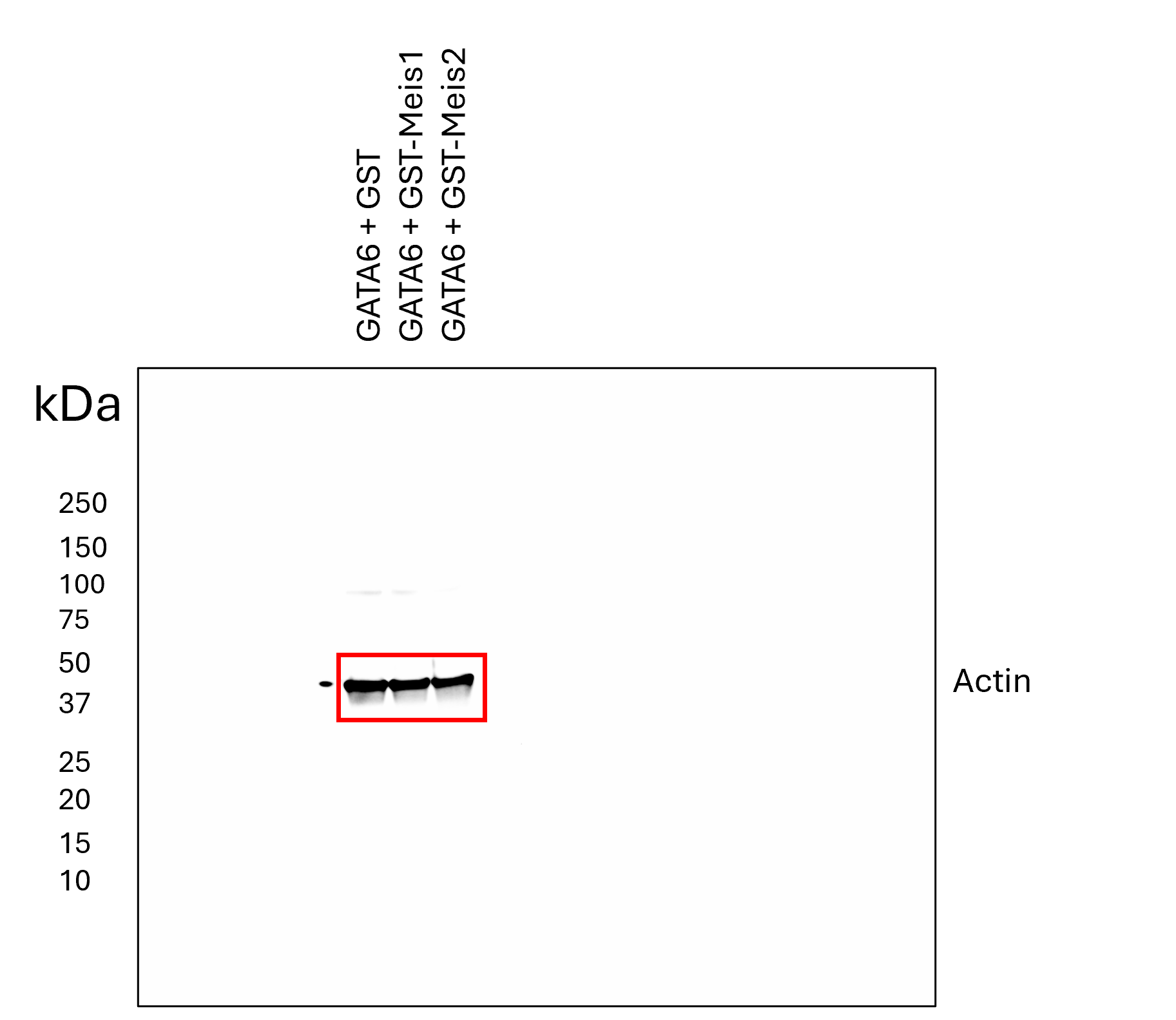

Supplement: Supplementary file 6 — Source data Fig. 4 [file 44318_2025_385_MOESM6_ESM.zip › Fig 4_Source_data/Source data Fig4H/western gata6 experiment actin input.tif]

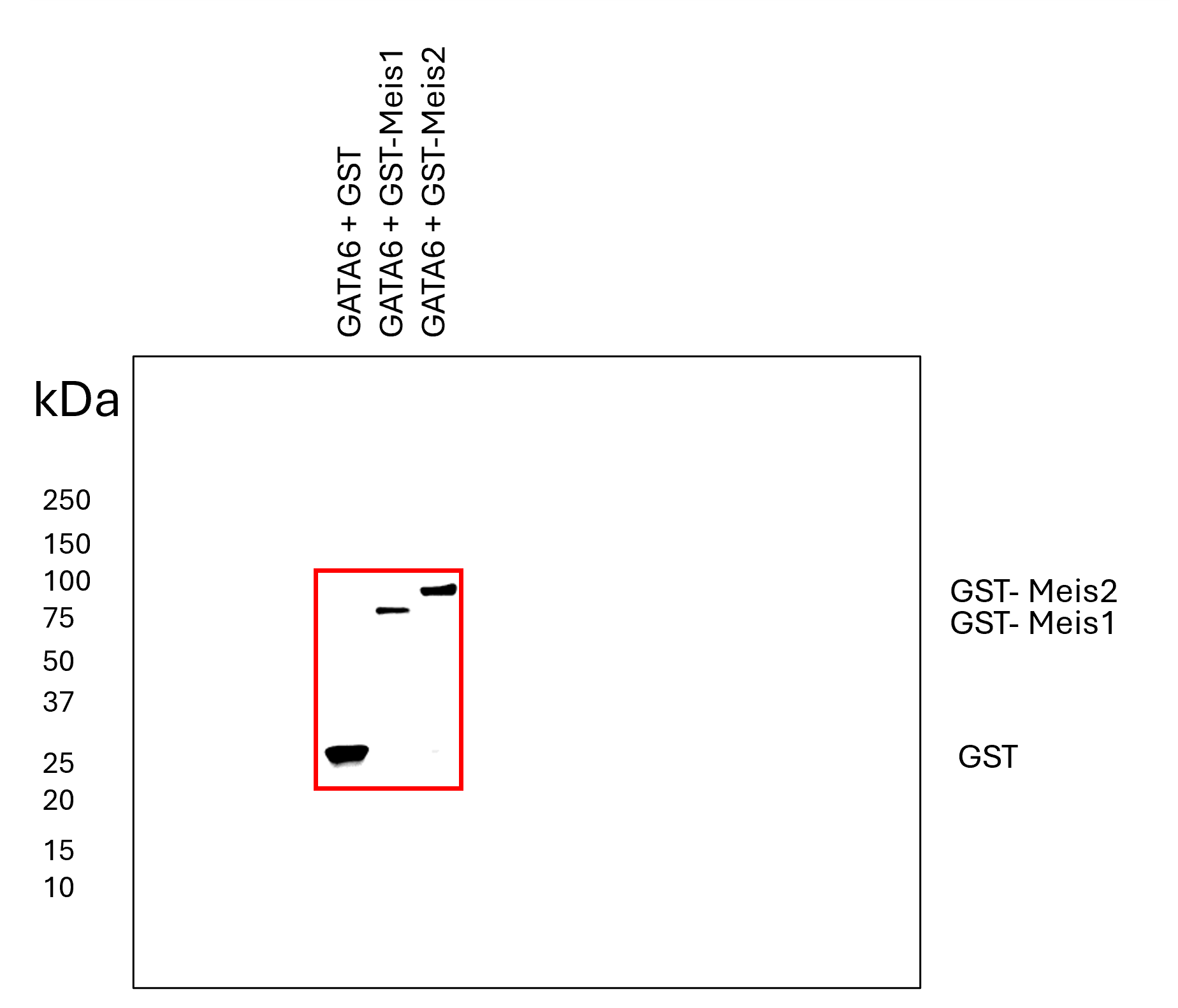

Supplement: Supplementary file 6 — Source data Fig. 4 [file 44318_2025_385_MOESM6_ESM.zip › Fig 4_Source_data/Source data Fig4H/western gata6 experiment gst input.tif]

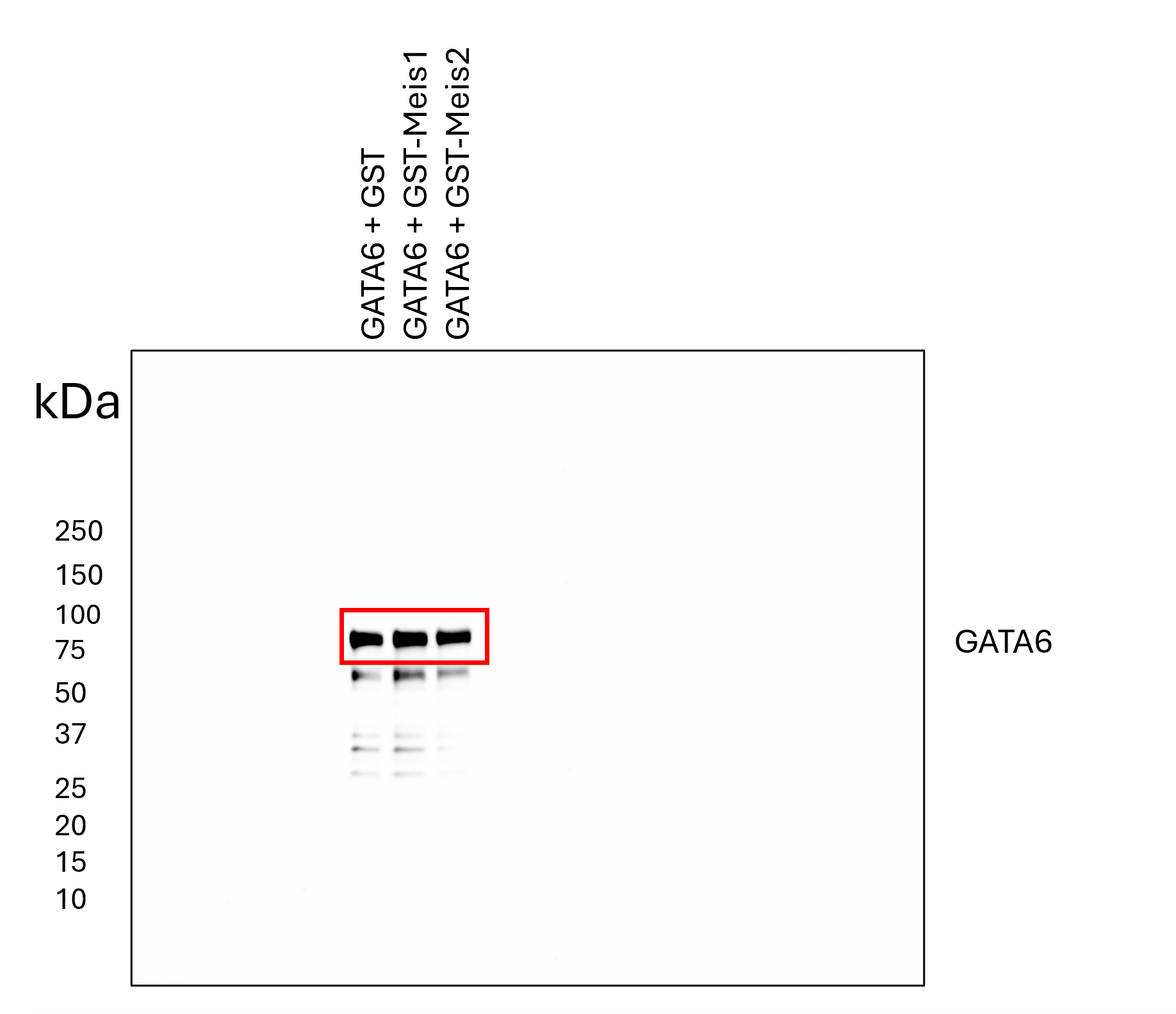

Supplement: Supplementary file 6 — Source data Fig. 4 [file 44318_2025_385_MOESM6_ESM.zip › Fig 4_Source_data/Source data Fig4H/western gata6 experiment gata6 input.tif]

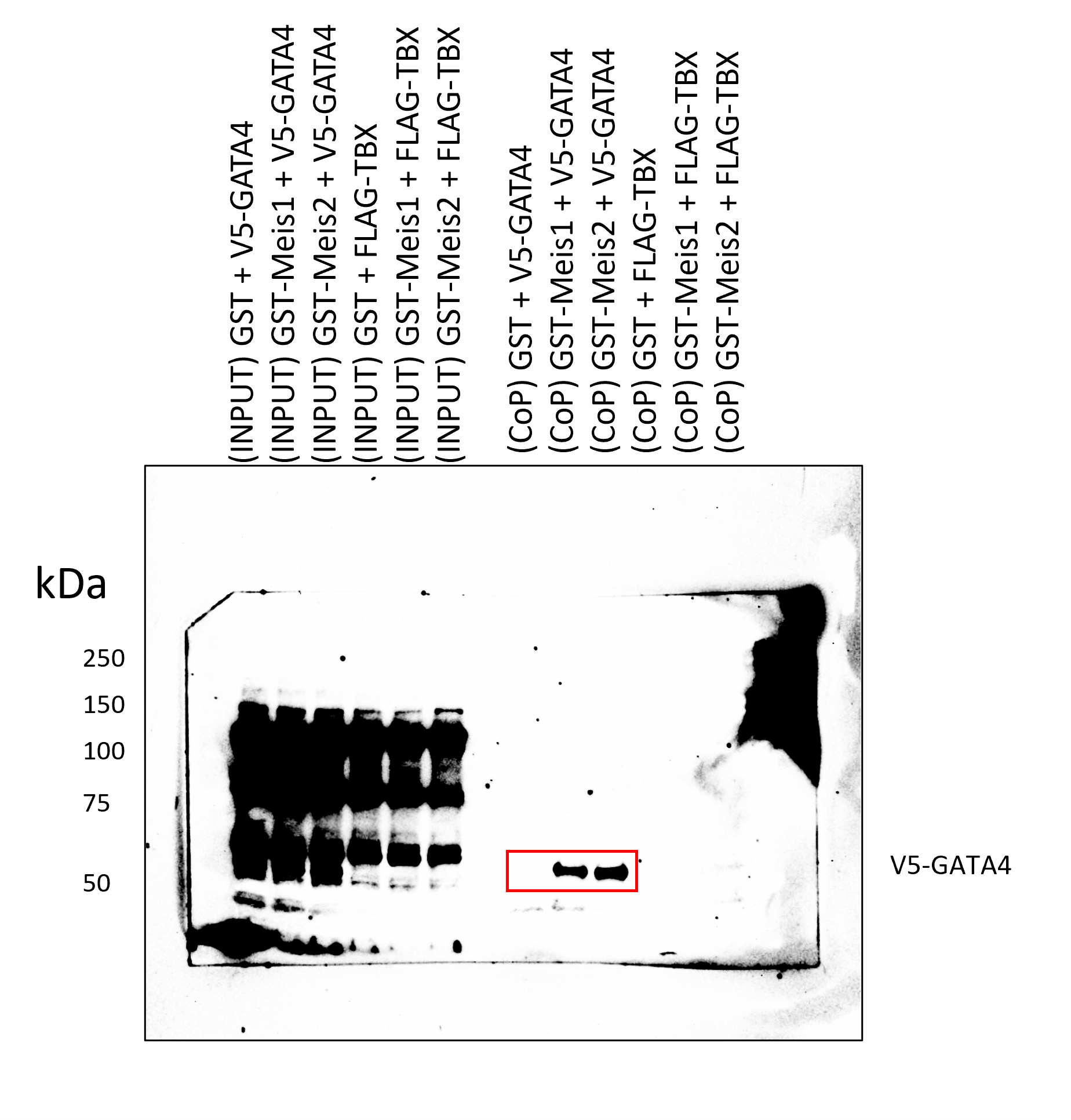

Supplement: Supplementary file 6 — Source data Fig. 4 [file 44318_2025_385_MOESM6_ESM.zip › Fig 4_Source_data/Source data Fig4H/western gata4 experiment Gata4 CoP.tif]

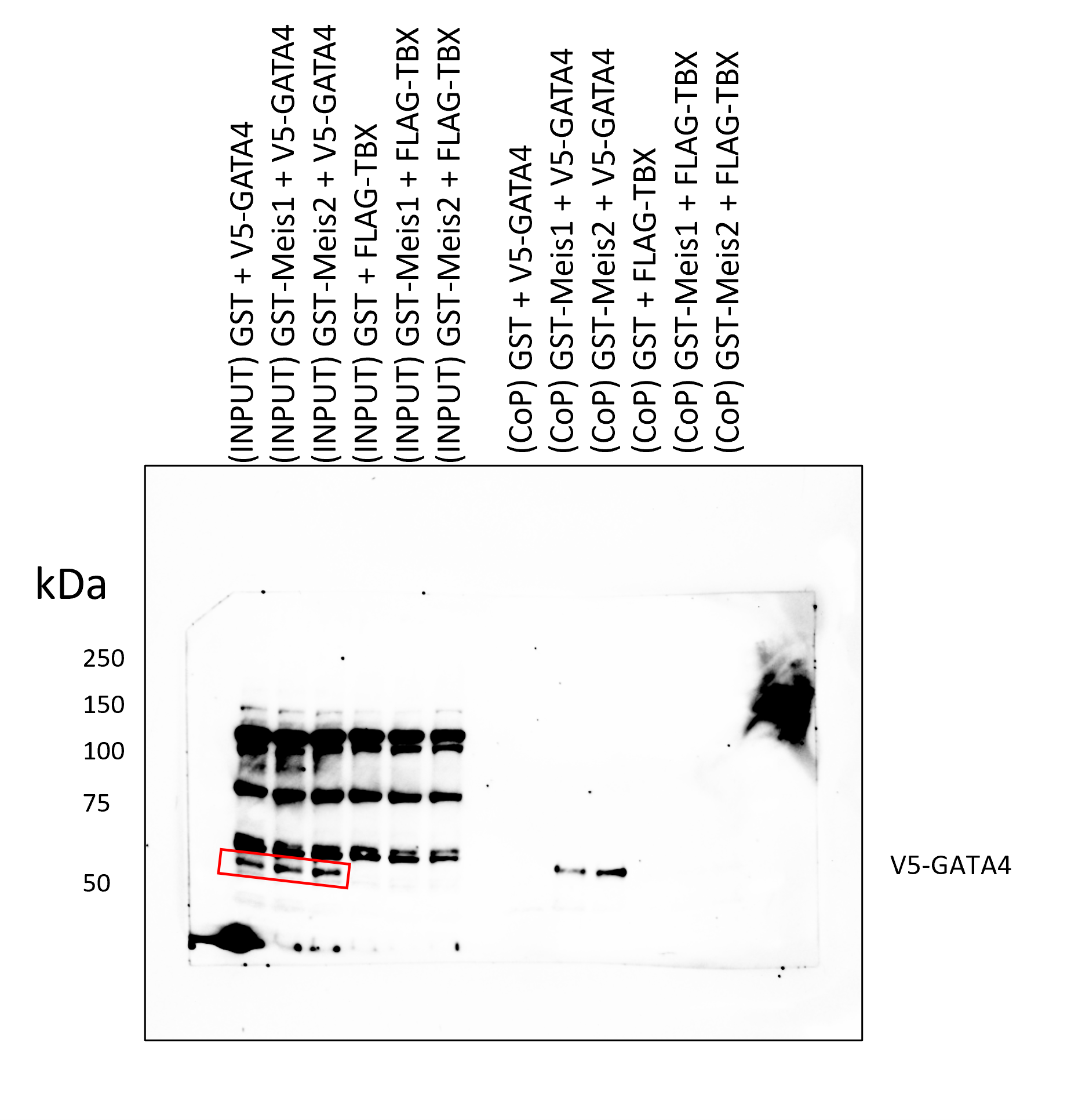

Supplement: Supplementary file 6 — Source data Fig. 4 [file 44318_2025_385_MOESM6_ESM.zip › Fig 4_Source_data/Source data Fig4H/western gata4 experiment gata4 input.tif]

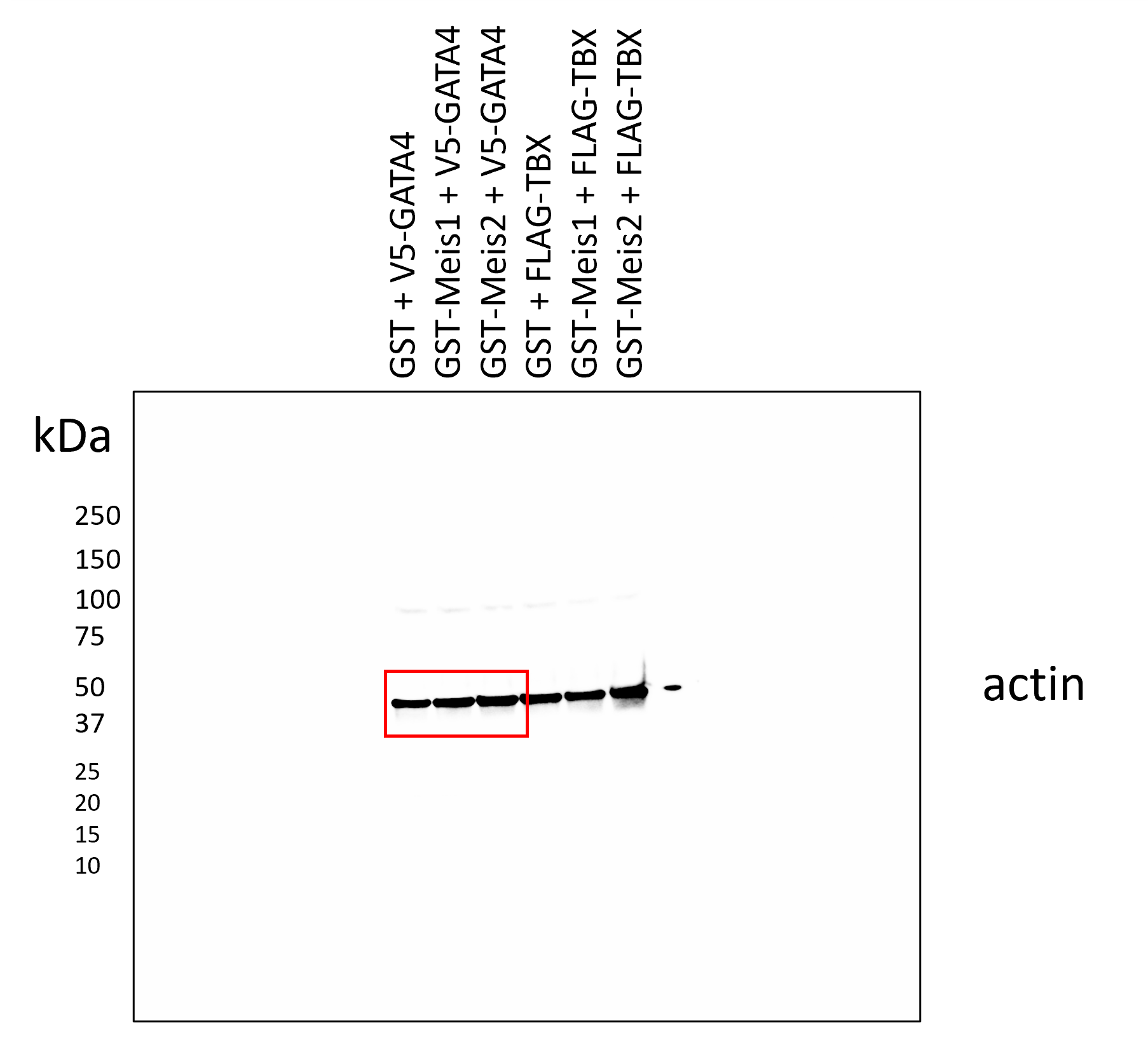

Supplement: Supplementary file 6 — Source data Fig. 4 [file 44318_2025_385_MOESM6_ESM.zip › Fig 4_Source_data/Source data Fig4H/western gata4 experiment actin input.tif]

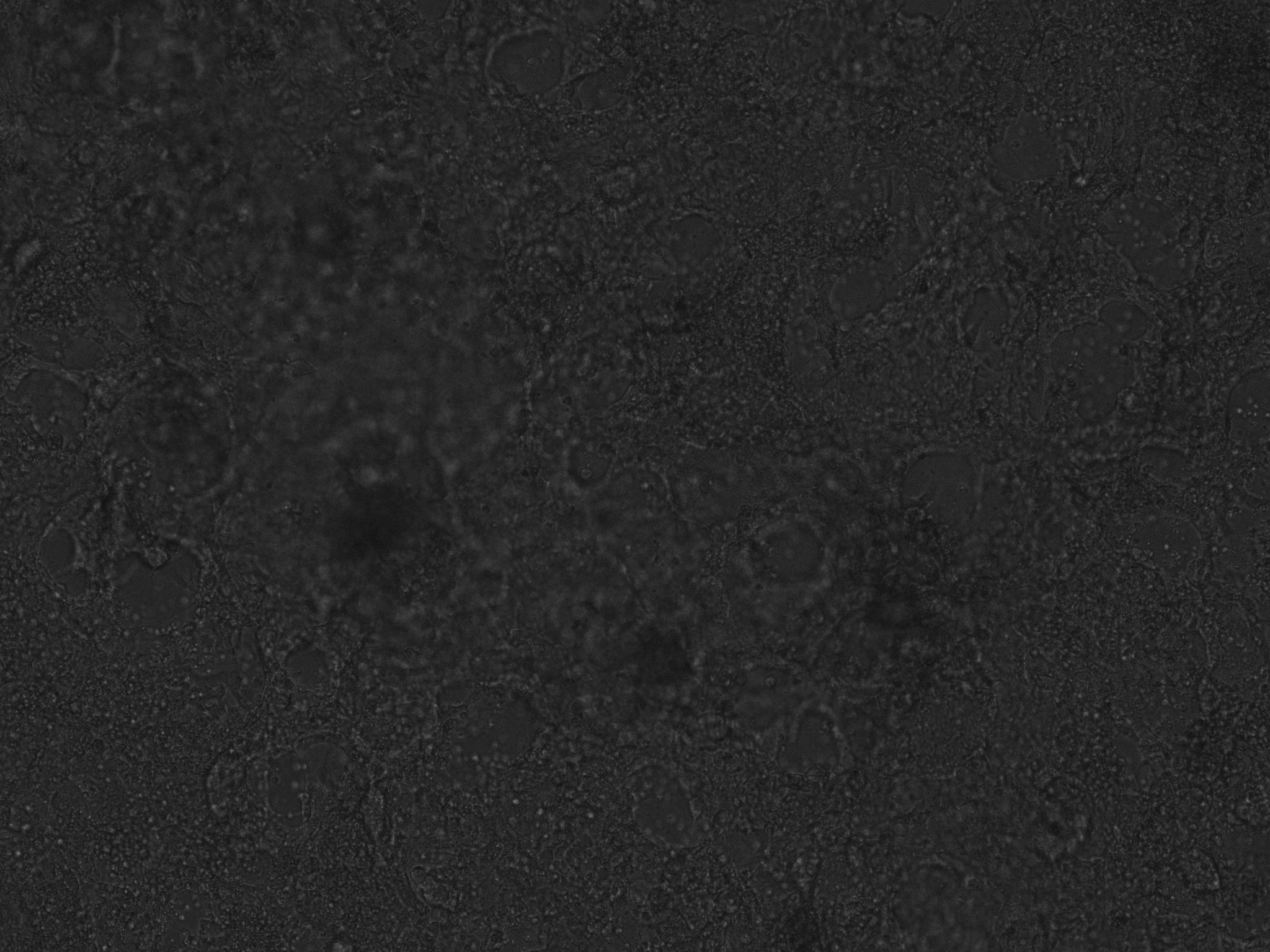

Supplement: Supplementary file 7 — Source data Fig. 5 [file 44318_2025_385_MOESM7_ESM.zip › Fig 5_Source_data/Source data Figure 5B/No dox_TRANS.tiff]

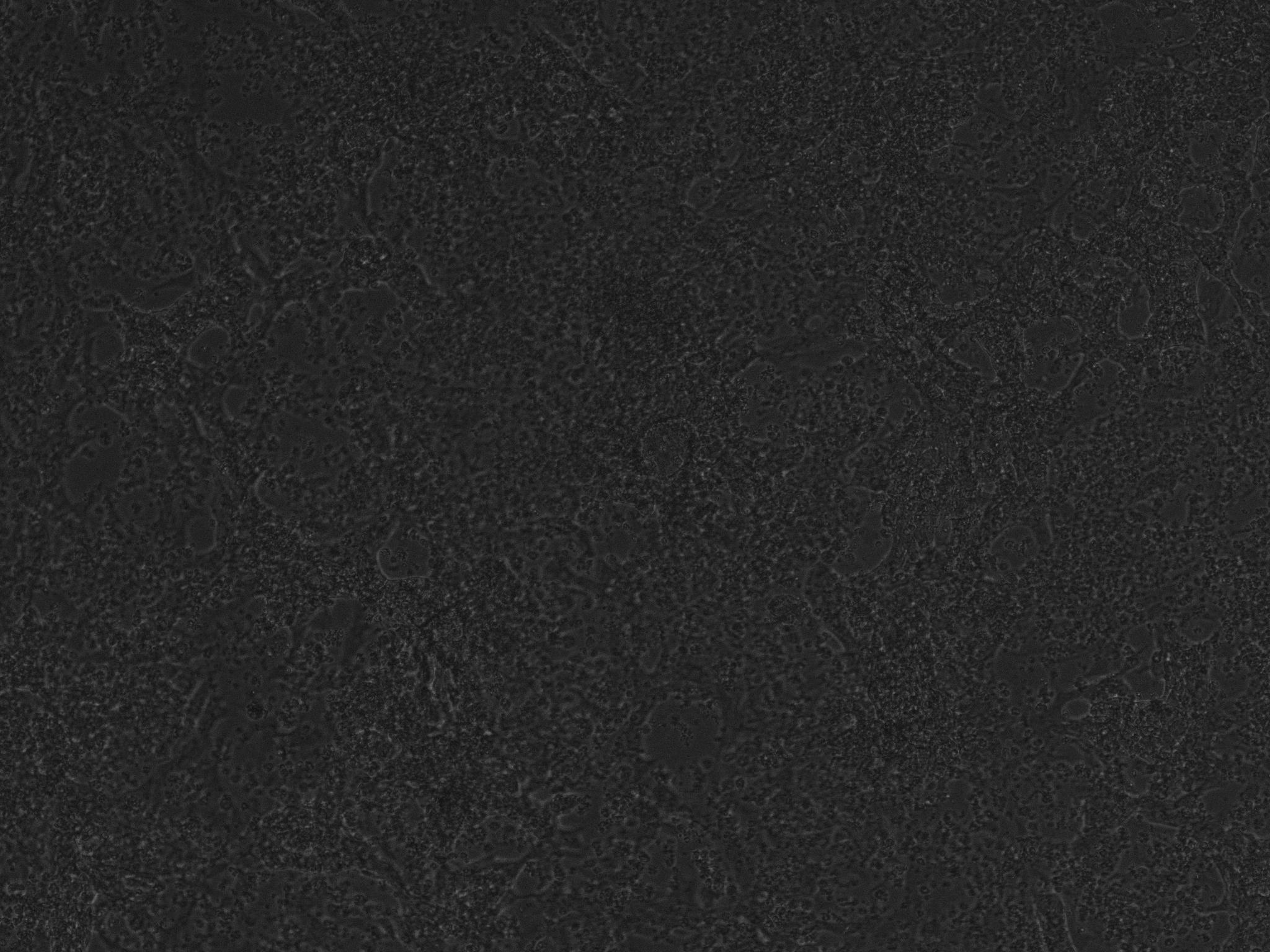

Supplement: Supplementary file 7 — Source data Fig. 5 [file 44318_2025_385_MOESM7_ESM.zip › Fig 5_Source_data/Source data Figure 5B/+ Dox_TRANS.tiff]

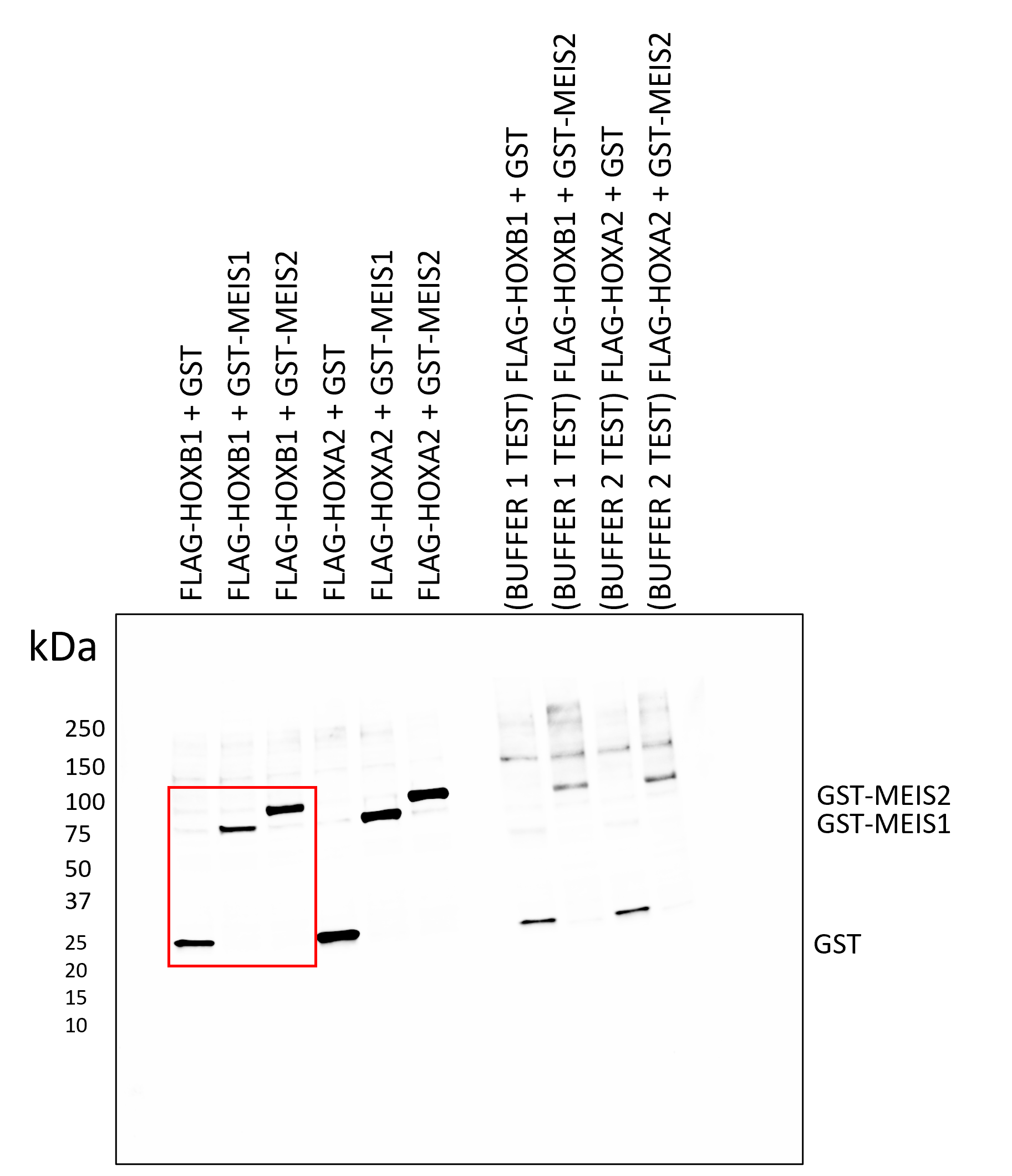

Supplement: Supplementary file 7 — Source data Fig. 5 [file 44318_2025_385_MOESM7_ESM.zip › Fig 5_Source_data/Source data Fig 5C/western gst input.tif]

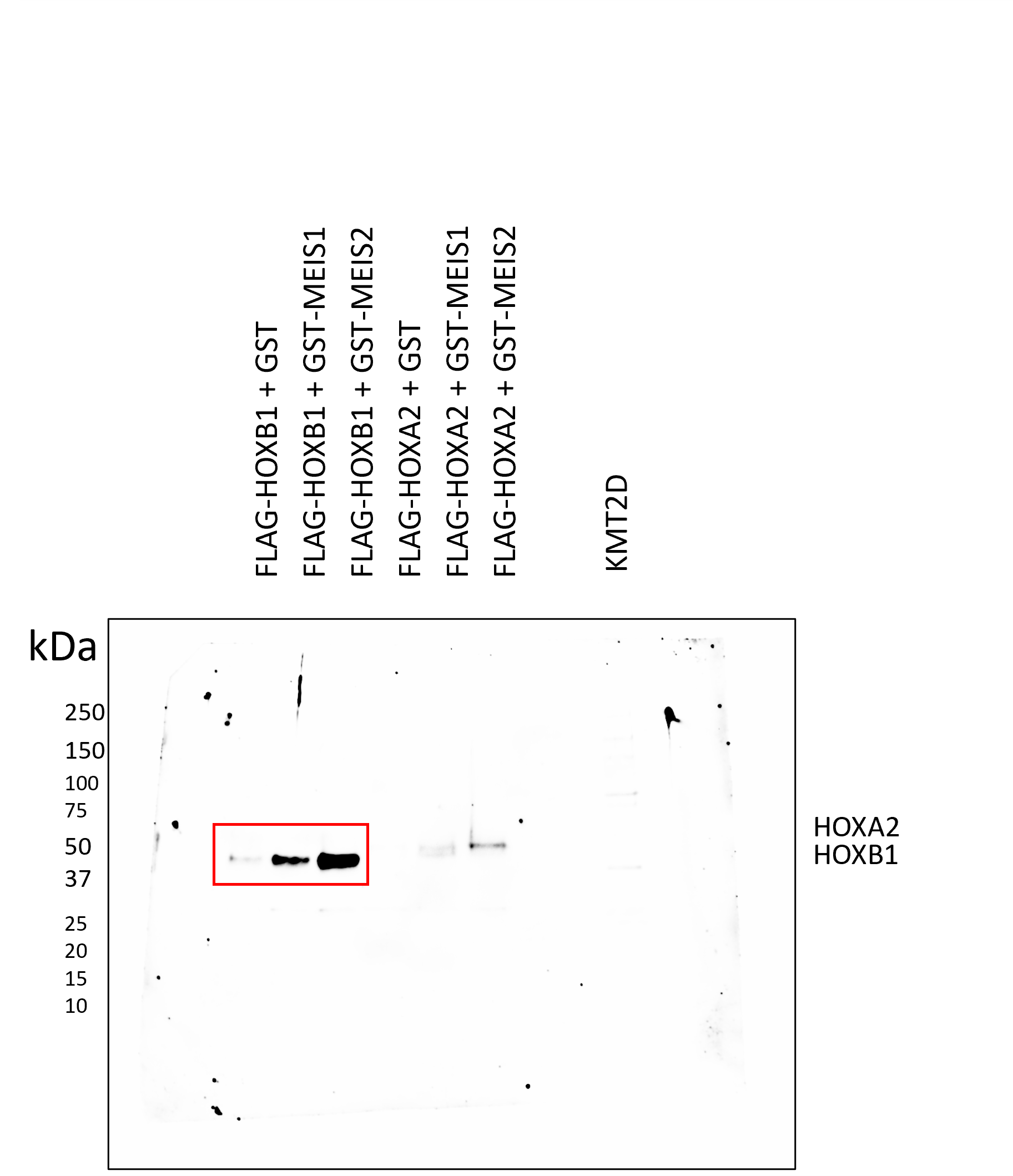

Supplement: Supplementary file 7 — Source data Fig. 5 [file 44318_2025_385_MOESM7_ESM.zip › Fig 5_Source_data/Source data Fig 5C/western flag CoP.tif]

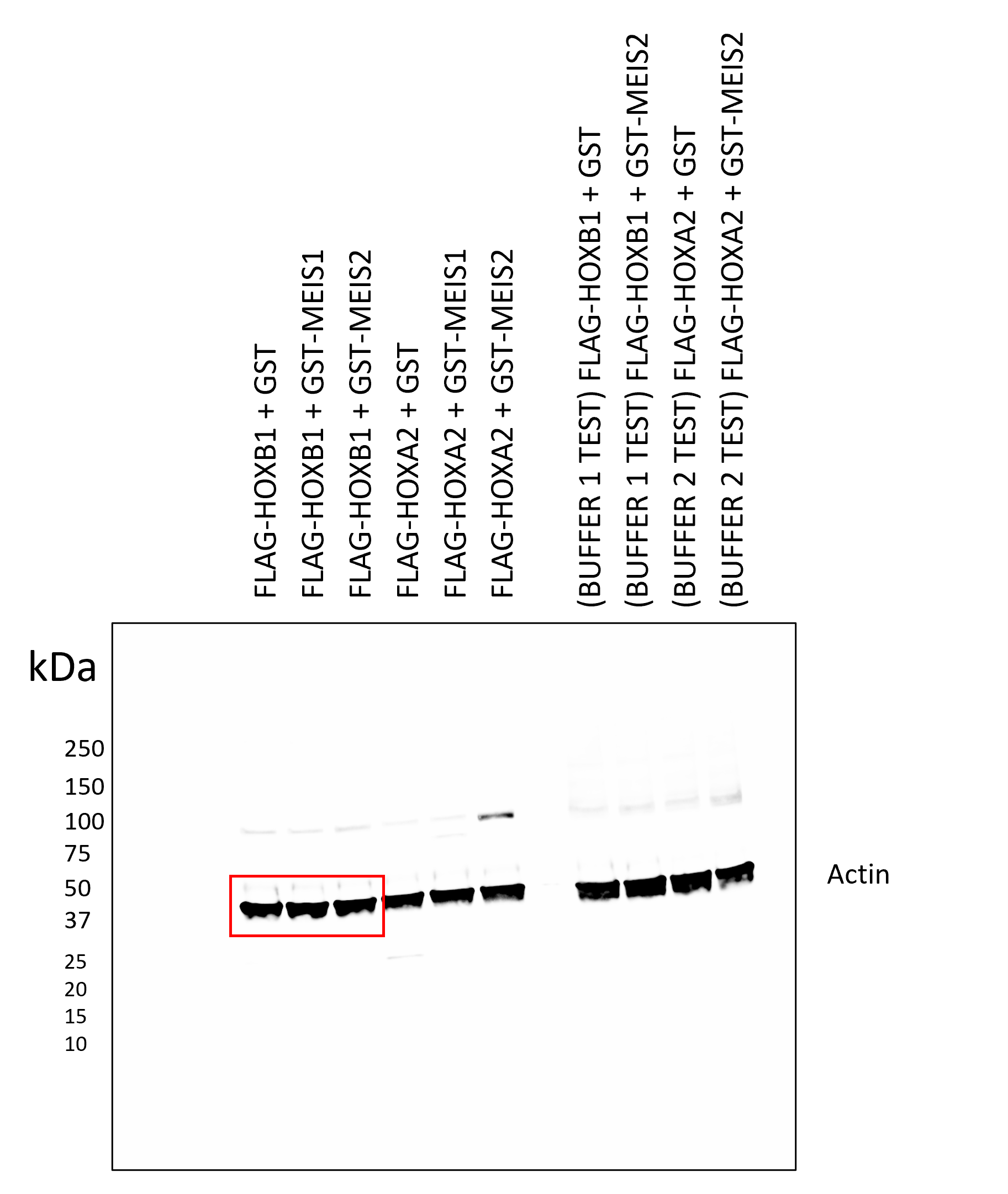

Supplement: Supplementary file 7 — Source data Fig. 5 [file 44318_2025_385_MOESM7_ESM.zip › Fig 5_Source_data/Source data Fig 5C/western actin input.tif]

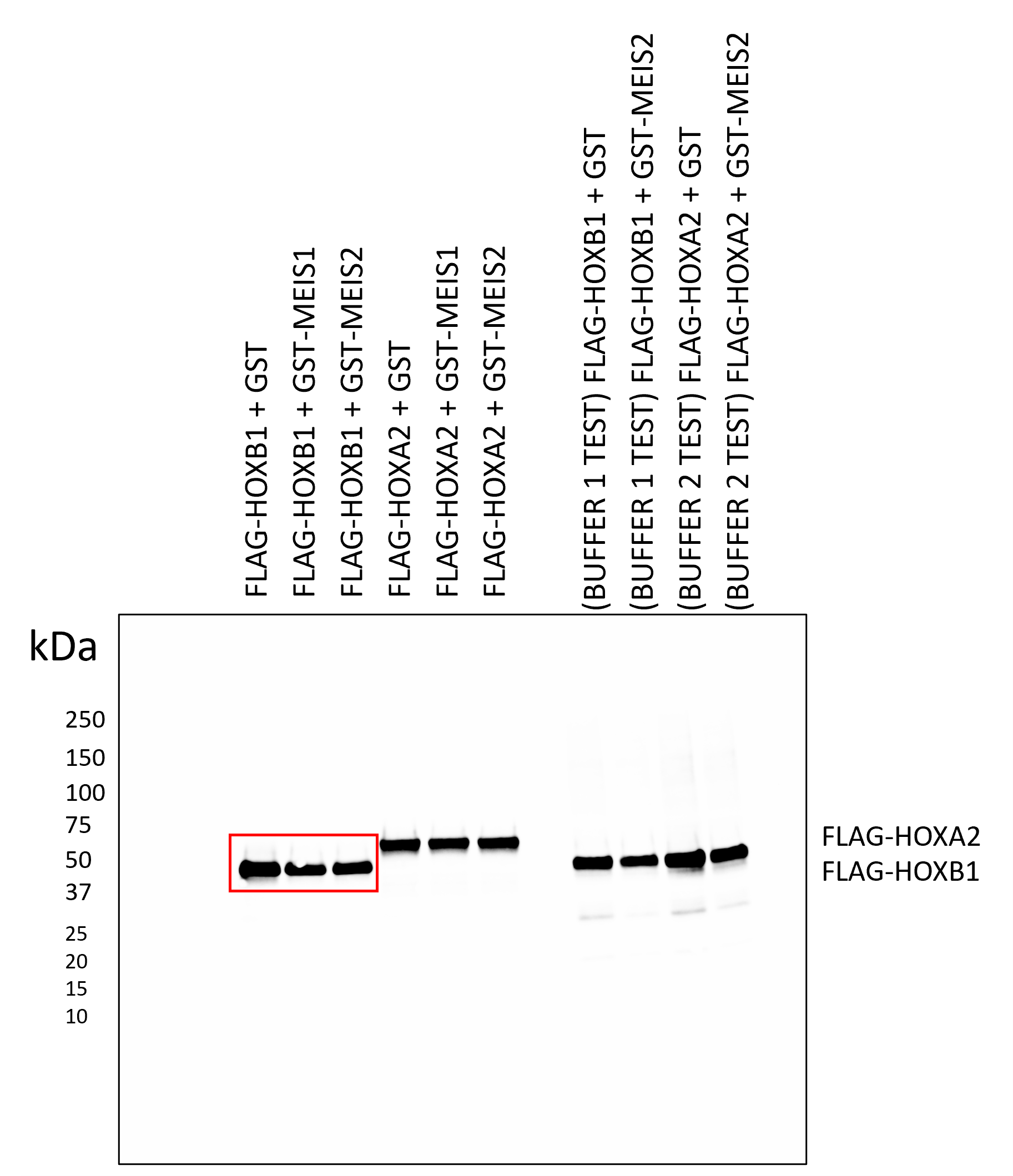

Supplement: Supplementary file 7 — Source data Fig. 5 [file 44318_2025_385_MOESM7_ESM.zip › Fig 5_Source_data/Source data Fig 5C/western flag input.tif]

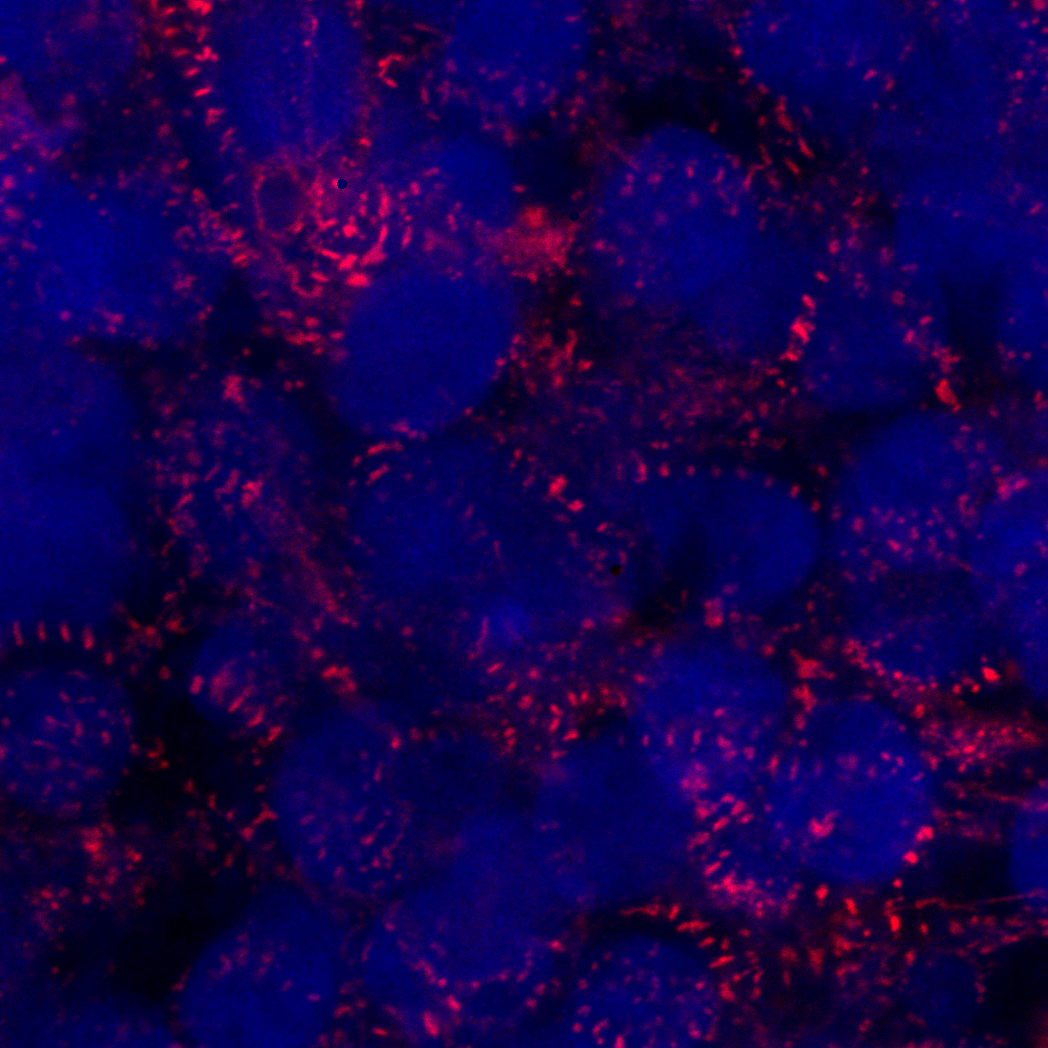

Supplement: Supplementary file 8 — Figure EV2 Source Data [file 44318_2025_385_MOESM8_ESM.zip › Source data Figure EV 2B/WT_ACTN2_Overlay_63x.tif]

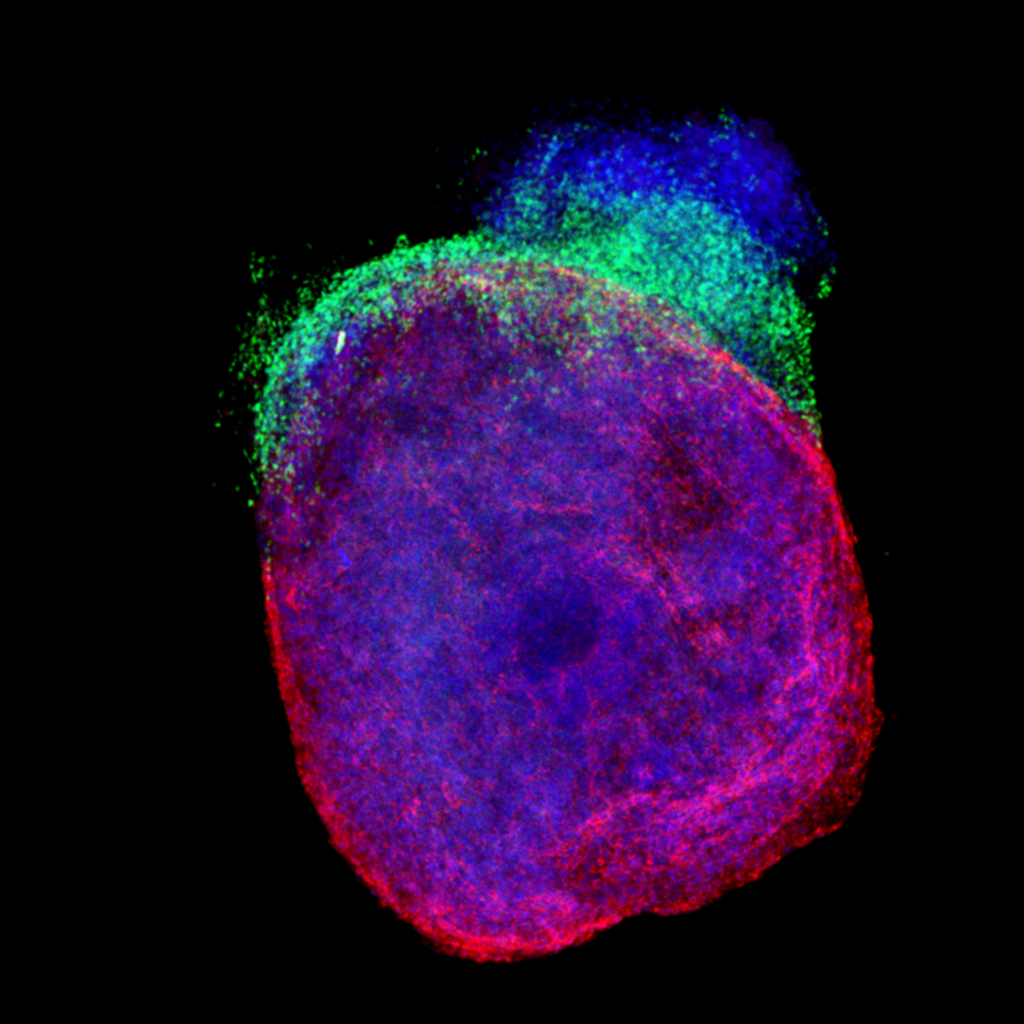

Supplement: Supplementary file 8 — Figure EV2 Source Data [file 44318_2025_385_MOESM8_ESM.zip › Source data Figure EV 2B/WT_all_Overlay_10x.tif]

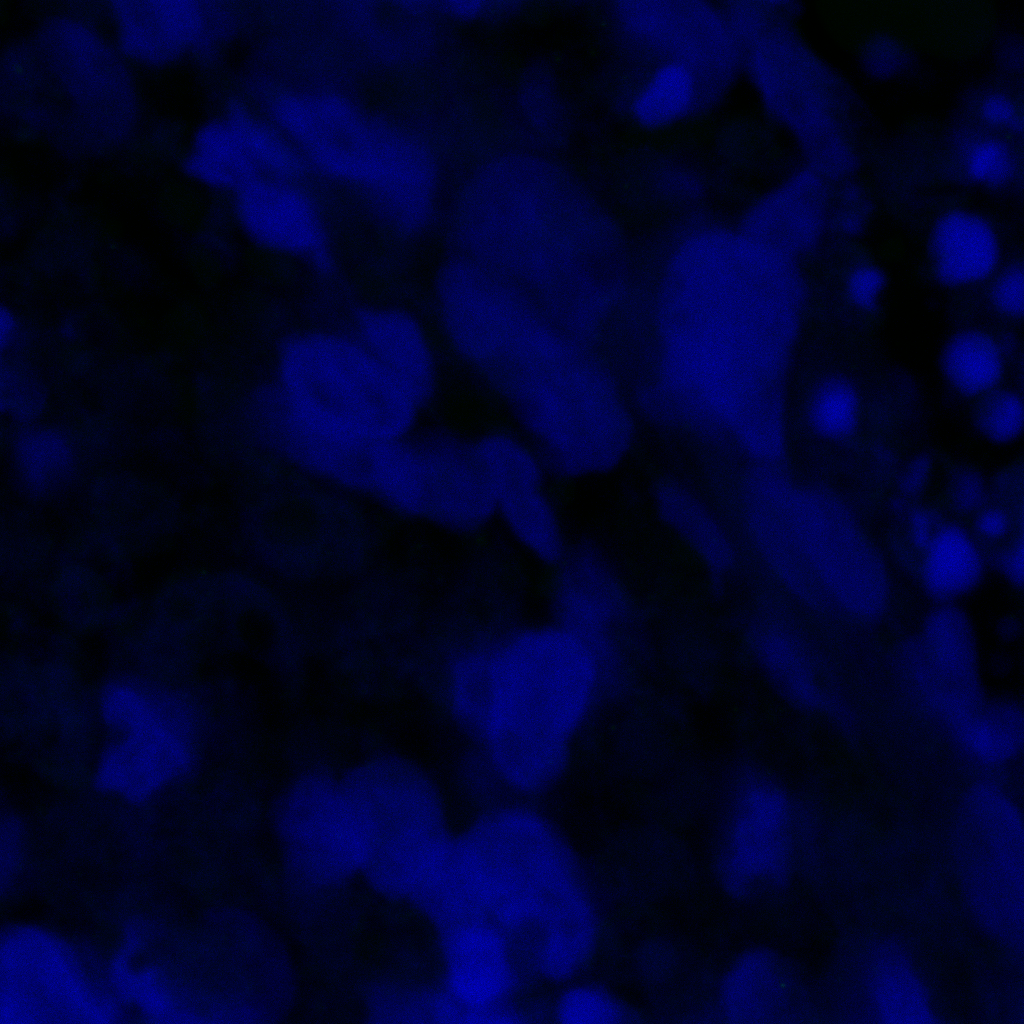

Supplement: Supplementary file 8 — Figure EV2 Source Data [file 44318_2025_385_MOESM8_ESM.zip › Source data Figure EV 2B/KO_WT1_Overlay_63x.tif]

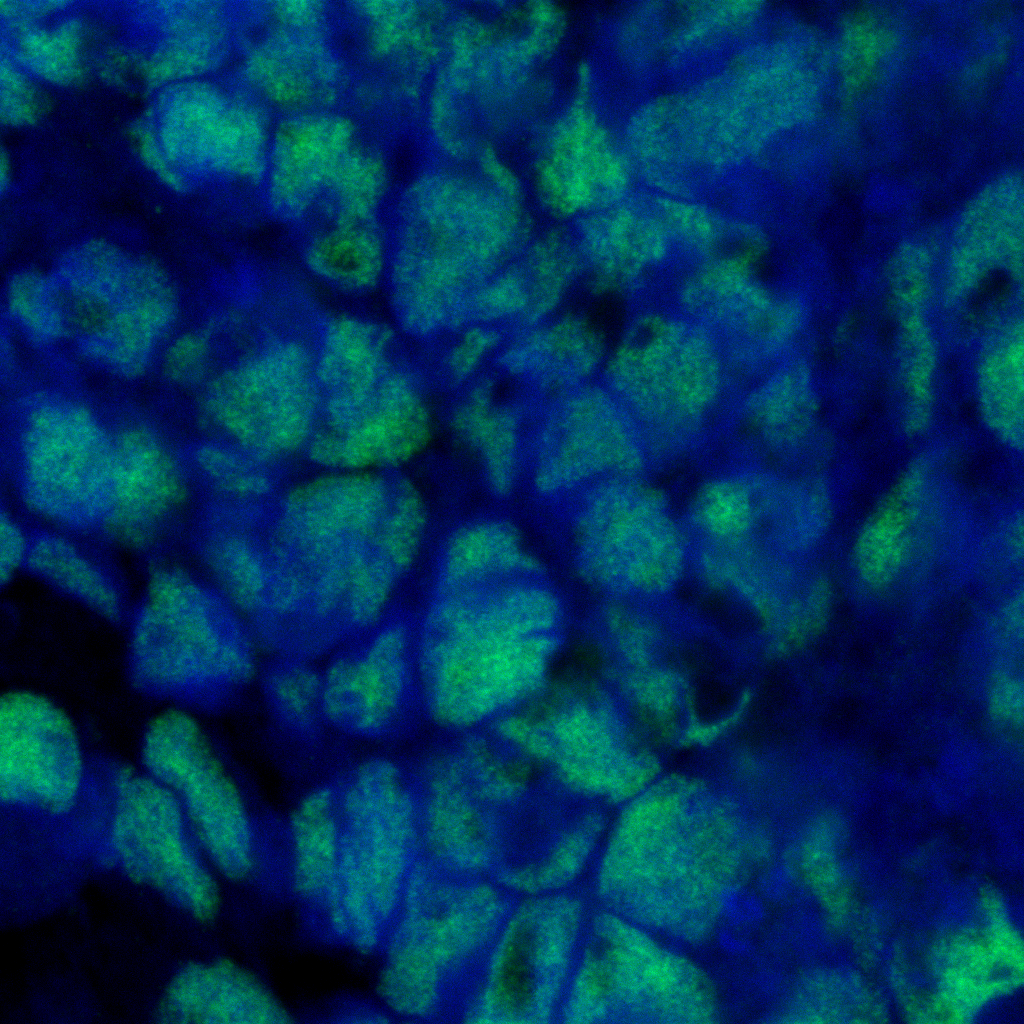

Supplement: Supplementary file 8 — Figure EV2 Source Data [file 44318_2025_385_MOESM8_ESM.zip › Source data Figure EV 2B/WT_WT1_Overlay_63x.tif]

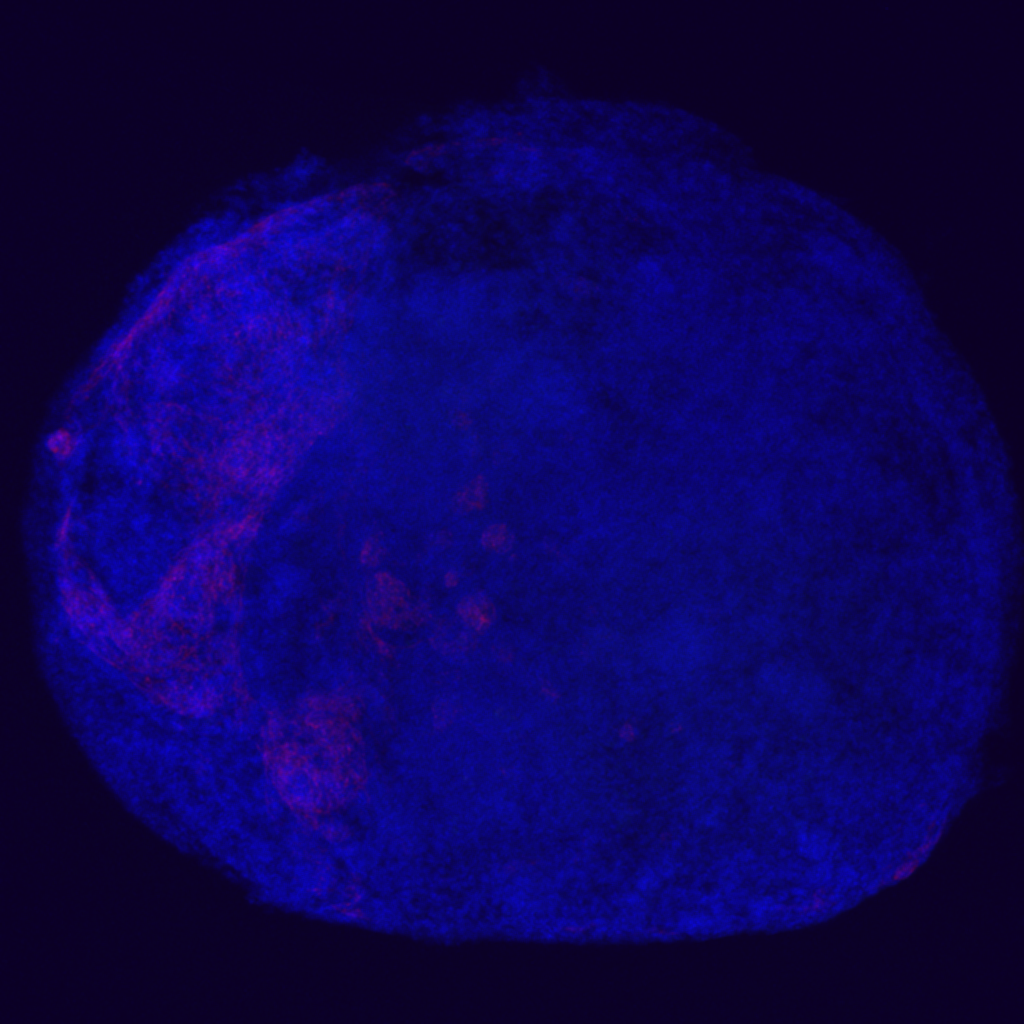

Supplement: Supplementary file 8 — Figure EV2 Source Data [file 44318_2025_385_MOESM8_ESM.zip › Source data Figure EV 2B/KO_Overlay_10x.tif]

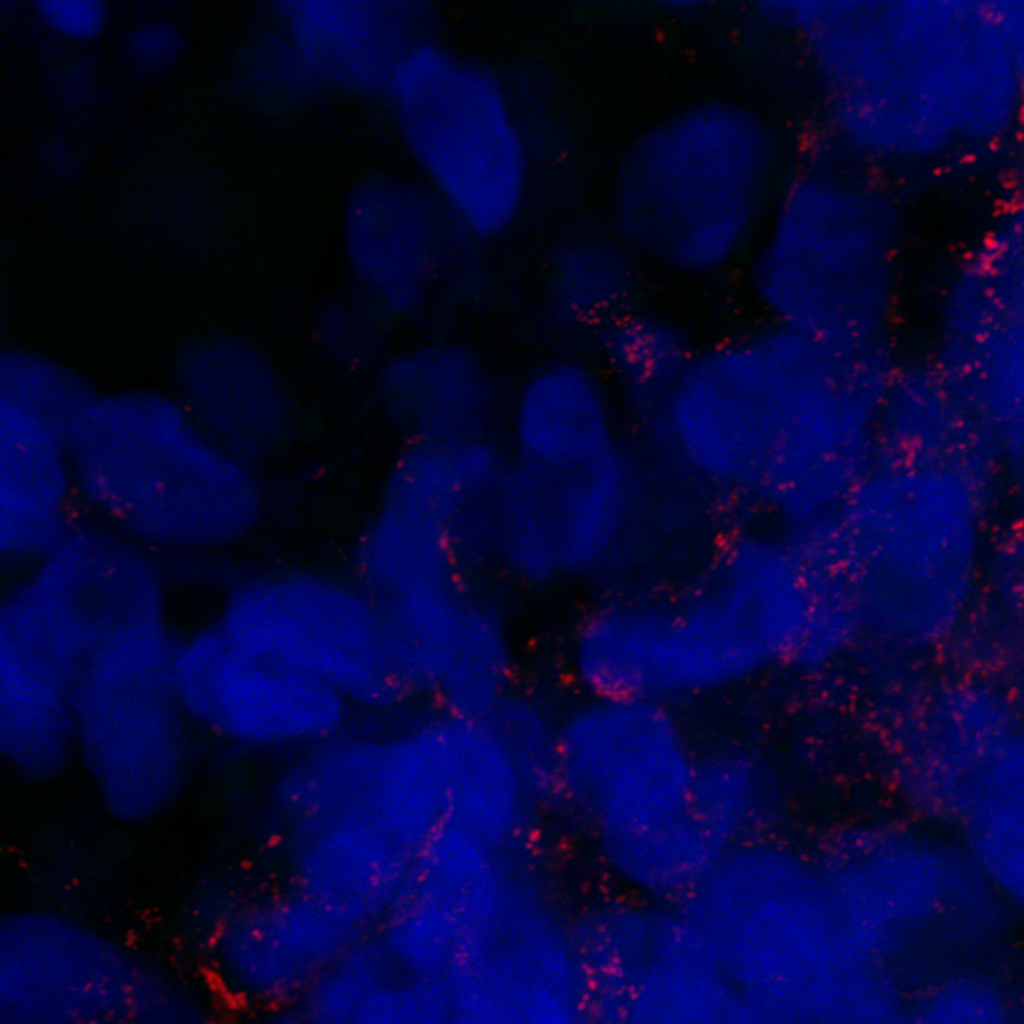

Supplement: Supplementary file 8 — Figure EV2 Source Data [file 44318_2025_385_MOESM8_ESM.zip › Source data Figure EV 2B/KO_ACTN2_Overlay_63x.tif]
