# Supplementary material for: Ubiquitous MEIS transcription factors actuate lineage-specific transcription to establish cell fate
Source: EMBO J. 2025 Feb 28;44(8):2232–62. doi: 10.1038/s44318-025-00385-5 (PMC12000411; doi:10.1038/s44318-025-00385-5)
Supplement: Supplementary file 8 — Figure EV2 Source Data [file 44318_2025_385_MOESM8_ESM.zip › Source data Figure EV 2B/Read me.docx]

Figure S2B is now Fig EV2C
