# Supplementary material for: Ubiquitous MEIS transcription factors actuate lineage-specific transcription to establish cell fate
Source: EMBO J. 2025 Feb 28;44(8):2232–62. doi: 10.1038/s44318-025-00385-5 (PMC12000411; doi:10.1038/s44318-025-00385-5)
Supplement: Supplementary file 9 — Expanded View Figures [file 44318_2025_385_MOESM9_ESM.pdf]

Expanded View Figures

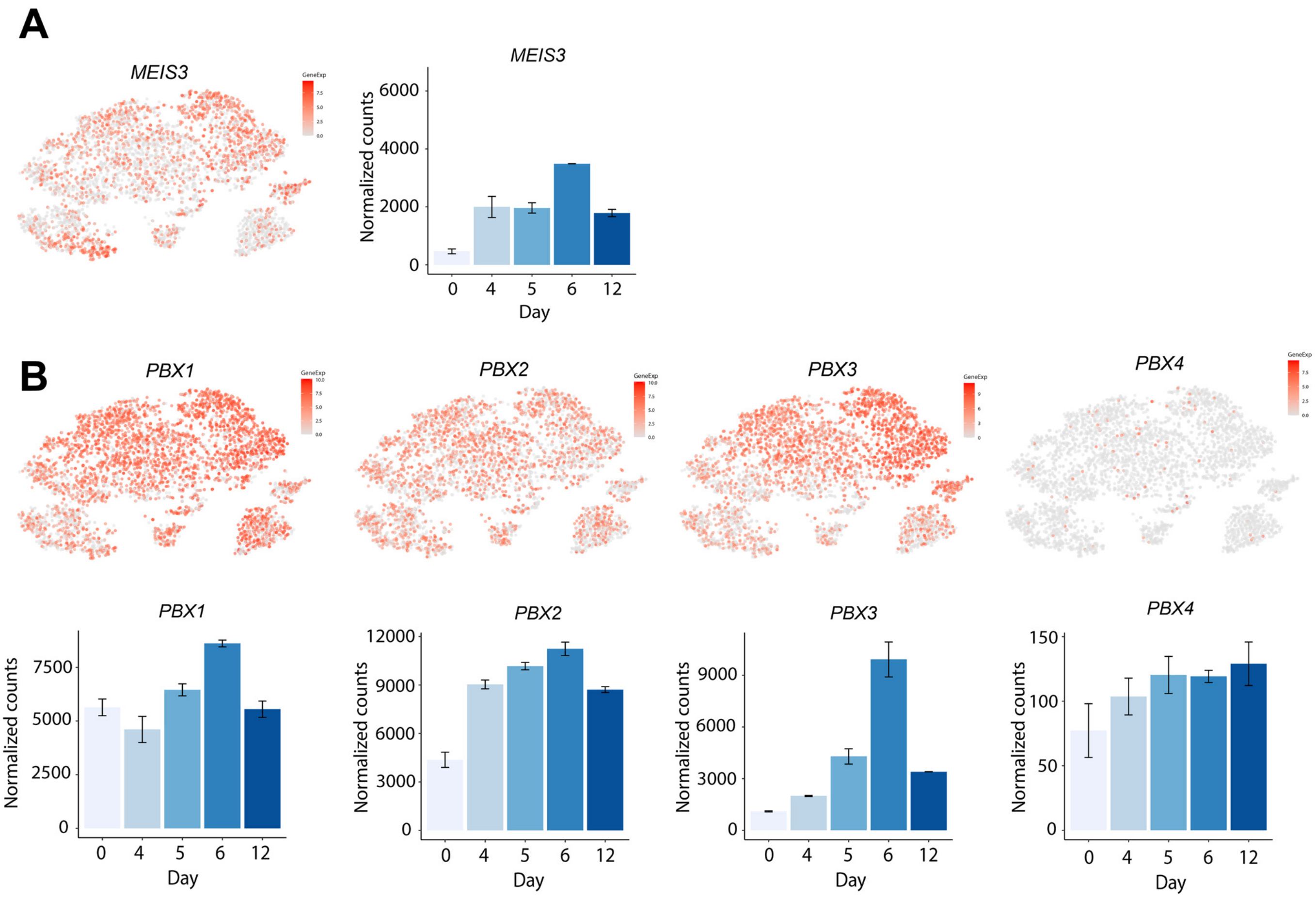

Q13

Figure EV1. (**A**, **B**) t-SNE plots showing gene expression level in the scRNA-seq cardiac differentiation time course, and corresponding bulk RNA-seq normalized counts by differentiation day, for *MEIS3* (**A**) and PBX family members (**B**). For bulk RNA-seq experiments, $n = 2$ (d0, d4, d5, d12); $n = 3$ (d6); the error bars indicate the standard deviation (SD). The scRNA-seq cell populations are annotated in Fig. 1D.

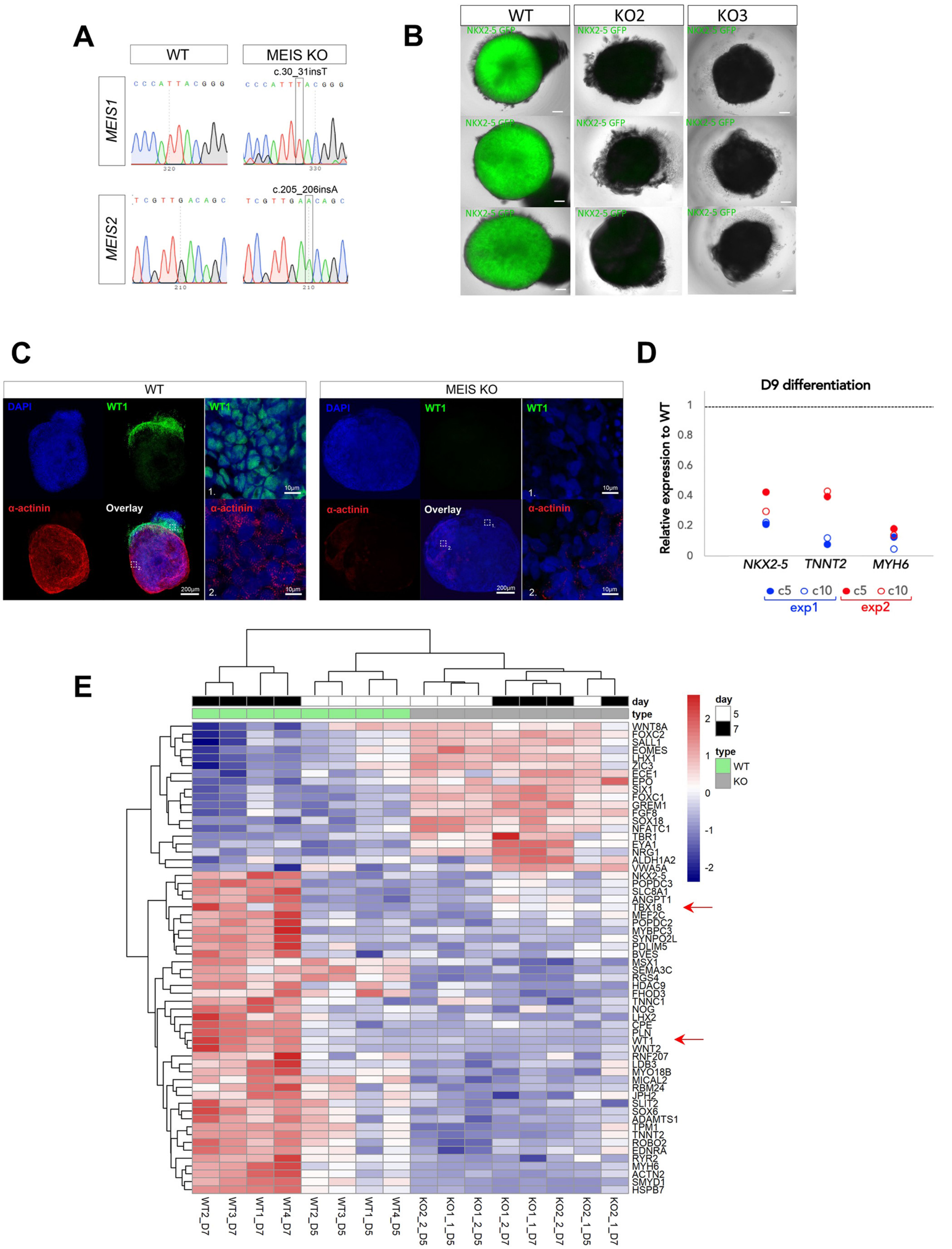
A
WT
MEIS KO
c.30_31insT
MEIS1
c.205_206insA
MEIS2
B
WT
KO2
KO3
NKX2-5 GFP
C
WT
MEIS KO
DAPI
WT1
α-actinin
Overlay
D
D9 differentiation
Relative expression to WT
NKX2-5
TNNT2
MYH6
c5
c10
exp1
exp2
E
day
type
WT
KO
WNT8A
FOXC2
SALL1
EOMES
LHX1
ZIC3
ECE1
EPO
SIX1
FOXC1
GREM1
FGF8
SOX18
NFATC1
TBR1
EYA1
NRG1
ALDH1A2
VWA5A
NKX2-5
POPDC3
SLC8A1
ANGPT1
TBX18
MEF2C
POPDC2
MYBPC3
SYNPO2L
PDLIM5
BVES
MSX1
SEMA3C
RGS4
HDAC9
FHOD3
TNNC1
NOG
LHX2
CPE
PLN
WT1
WNT2
RNF207
LDB3
MYO18B
MICAL2
RBM24
JPH2
SLIT2
SOX6
ADAMTS1
TPM1
TNNT2
ROBO2
EDNRA
RYR2
MYH6
ACTN2
SMYD1
HSPB7
WT2_D7
WT3_D7
WT1_D7
WT4_D7
WT2_D5
WT3_D5
WT1_D5
WT4_D5
KO2_2_D5
KO1_1_D5
KO1_2_D5
KO1_2_D7
KO1_1_D7
KO2_2_D7
KO2_1_D5
KO2_1_D7

 © The Author(s)

Figure EV2. (**A**) Sanger sequencing chromatograms showing the presence of homozygous insertion frameshift-causing mutations in *MEIS1* (c.30_31insT) and *MEIS2* (c.205_206insA) of the MEIS KO clones KO1, KO2 and KO3. The reference wild-type sequencing is also shown. (**B**) Live imaging of cardiac lineage reporter *NKX2-5-GFP* in wild-type and two additional MEIS KO EBs at d12 (KO2 and KO3 clones). In the same conditions, MEIS KO EBs fail to activate *NKX2-5* expression. The GFP channel is overlayed on the brightfield images. Scale bar = 200 μm. (**C**) Confocal microscopy image of WT1 (epicardial cells, green), α-actinin (cardiomyocytes, red) and nuclei (DAPI, blue) in day 12 EBs generated from wild-type and MEIS KO hESCs. The high magnification inserts show a sarcomere staining pattern with α-actinin and nuclear WT1, both of which are absent in the MEIS KO line. (**D**) Downregulation of cardiomyocyte markers in d9 MEIS KO MAN13 cells. Analysis of two independent clones (C5 and C10) with MEIS1-2 knockouts generated in a single round in the hESC line MAN13. The expression levels of *NKX2-5*, *TNNT2*, and *MYH6* were measured by qPCR in two independent differentiation experiments and are presented as percentages relative to the expression levels observed in wild-type (WT) cells. (**E**) Expression of cardiac mesoderm GO genes in wild-type (green) and MEIS KO (gray) cells at d5 (white) and d7(black). Top 60 genes with significant changes in expression at d7 (log FC > [1]; *P*adj <0.05) are plotted. The epicardial markers *TBX18* and *WT1* are highlighted by red arrows. Source data are available online for this figure.

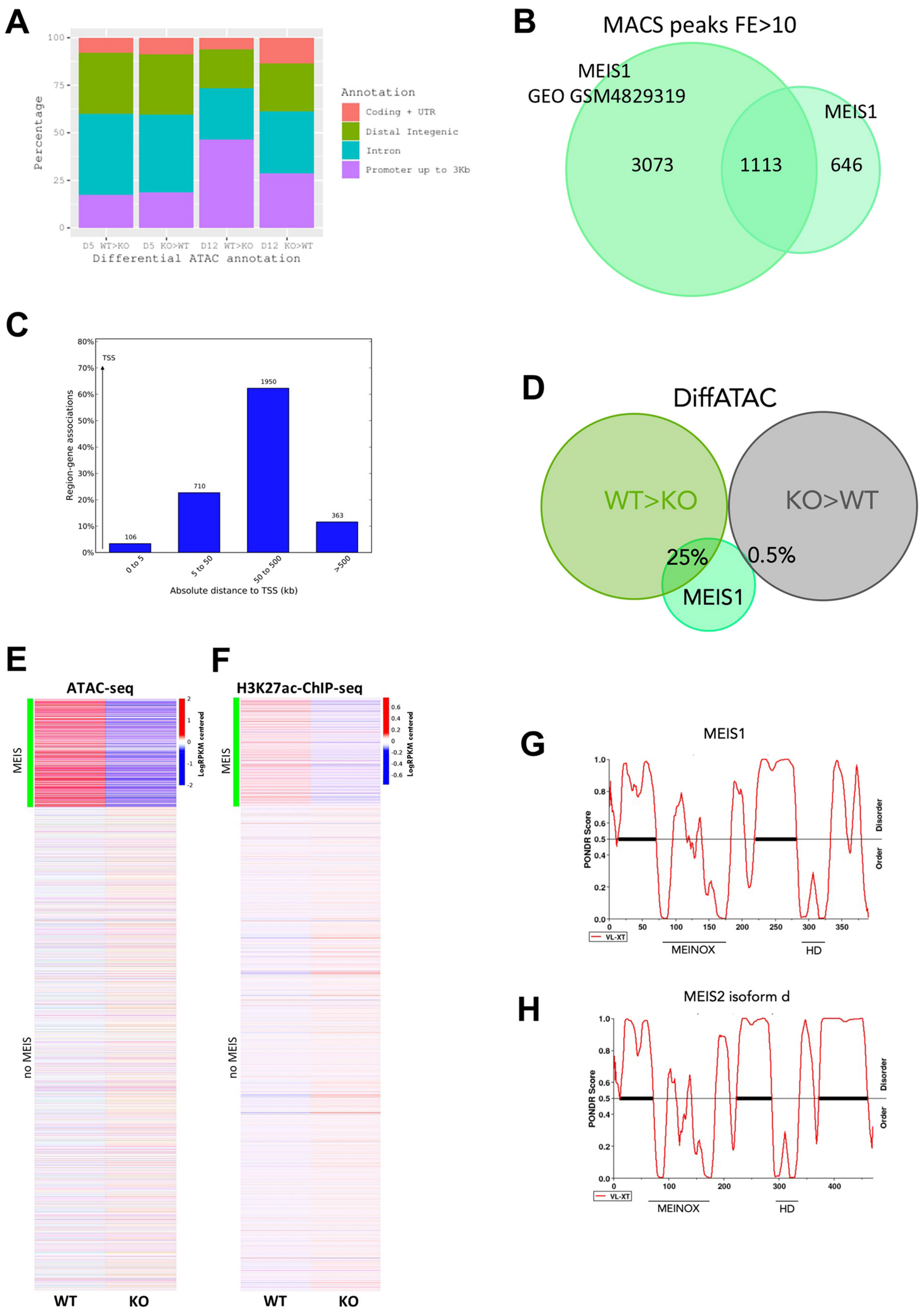
A
Percentage
Annotation
Coding + UTR
Distal Integenic
Intron
Promoter up to 3Kb
D5 WT>KO
D5 KO>WT
D12 WT>KO
D12 KO>WT
Differential ATAC annotation
B
MACS peaks FE>10
MEIS1
GEO GSM4829319
MEIS1
3073
1113
646
C
TSS
Region-gene associations
106
710
1950
363
0 to 5
5 to 50
50 to 500
>500
Absolute distance to TSS (kb)
D
DiffATAC
WT>KO
KO>WT
25%
0.5%
MEIS1
E
ATAC-seq
MEIS
no MEIS
LogRPKM centered
WT
KO
F
H3K27ac-ChIP-seq
MEIS
no MEIS
LogRPKM centered
WT
KO
G
MEIS1
PONDR Score
Disorder
Order
VL-XT
MEINOX
HD
H
MEIS2 isoform d
PONDR Score
Disorder
Order
VL-XT
MEINOX
HD

 © The Author(s)

Figure EV3. (**A**) Genomic distribution of differential chromatin accessibility in wild-type and MEIS1-2 KO at d5 and d12. At d5, differential accessibility between wild-type and mutant is largely observed at distal regions and introns. At d12, promoters make half of the differentially accessible regions in wild-type while in the absence of MEIS1-2, distal regions and introns remain more highly accessible. (**B**) Comparison of MEIS1 ChIP-seq with MEIS1 ChIP-seq in d6 cardiac progenitors (GEO accession: GSM4829319). MACS peaks intersection shows that >60% of our MEIS1 peaks (FE > 10) is replicated in the GSM4829319 dataset. The overlap is 52%, when considering all MACS peaks (no enrichment cutoff). (**C**) Genomic distribution of top FE > 10 MEIS1 peaks. (**D**) Intersection of top FE > 10 MEIS1 peaks with differential ATAC-seq peaks. High-confidence MEIS1 binding overlaps almost exclusively with chromatin that is more accessible in wild-type conditions (25% of top MEIS1 peaks; $n = 446$), indicating a direct association between MEIS1 binding and accessible chromatin. In contrast, in the mutant, chromatin accessibility is independent of MEIS, with only 0.5% of top MEIS peaks ($n = 8$) intersecting ATAC peaks higher in MEIS KO. This suggests that MEIS does not directly establish repressive chromatin environments; rather, sites that become more accessible in the absence of MEIS are likely opened by upregulated TFs. (**E**) ATAC-seq average logRPKM values across replicates, mean-centered across wild-type and MEIS KO samples. Of 142,411 total open chromatin regions, 26,062 are occupied by MEIS1. (**F**) H3K27ac ChIP-seq average logRPKM values across replicates, mean-centered across wild-type and MEIS KO samples. Short reads were counted in open chromatin regions; the number of regions is as in (**E**). (**G**, **H**) Output of PONDR (http://www.pondr.com) (Romero et al, 1997) for MEIS1 and MEIS2 (isoform d). MEIS1-2 contain a majority of IDRs and two well-conserved structured domains, the MEINOX domain and the homeodomain (Schulte and Geerts, 2019). The disordered C-terminal domain contains MEIS1 activation domain (Bisaillon et al, 2011; Mamo et al, 2006).

© The Author(s)

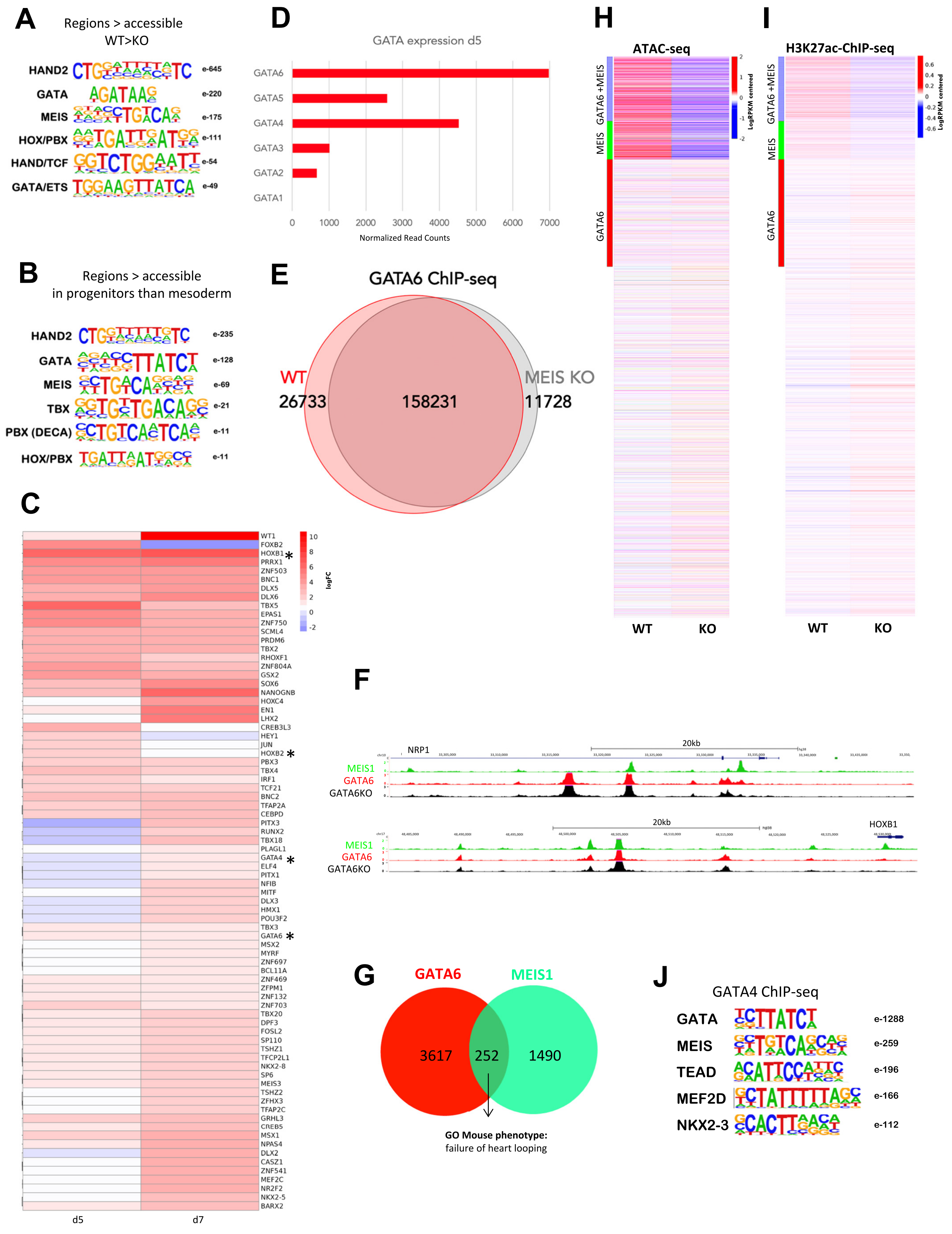
A
Regions > accessible WT>KO
HAND2 e-645
GATA e-220
MEIS e-175
HOX/PBX e-111
HAND/TCF e-54
GATA/ETS e-49
B
Regions > accessible in progenitors than mesoderm
HAND2 e-235
GATA e-128
MEIS e-69
TBX e-21
PBX (DECA) e-11
HOX/PBX e-11
C
WT1
FOXB2
HOXB1 *
PRRX1
ZNF503
BNC1
DLX5
DLX6
TBX5
EPAS1
ZNF750
SCML4
PRDM6
TBX2
RHOXF1
ZNF804A
GSX2
SOX6
NANOGNB
HOXC4
EN1
LHX2
CREB3L3
HEY1
JUN
HOXB2 *
PBX3
TBX4
IRF1
TCF21
BNC2
TFAP2A
CEBPD
PITX3
RUNX2
TBX18
PLAGL1
GATA4 *
ELF4
PITX1
NFIB
MITF
DLX3
HMX1
POU3F2
TBX3
GATA6 *
MSX2
MYRF
ZNF697
BCL11A
ZNF469
ZFPM1
ZNF132
ZNF703
TBX20
DPF3
FOSL2
SP110
TSHZ1
TFCP2L1
NKX2-8
SP6
MEIS3
TSHZ2
ZFHX3
TFAP2C
GRHL3
CREB5
MSX1
NPAS4
DLX2
CASZ1
ZNF541
MEF2C
NR2F2
NKX2-5
BARX2
d5
d7
logFC
10 8 6 4 2 0 -2
D
GATA expression d5
GATA6
GATA5
GATA4
GATA3
GATA2
GATA1
0 1000 2000 3000 4000 5000 6000 7000
Normalized Read Counts
E
GATA6 ChIP-seq
WT 26733
158231
MEIS KO 11728
F
NRP1
20kb
MEIS1
GATA6
GATA6KO
20kb
HOXB1
MEIS1
GATA6
GATA6KO
G
GATA6
MEIS1
3617
252
1490
GO Mouse phenotype: failure of heart looping
H
ATAC-seq
GATA6 +MEIS
MEIS
GATA6
LogRPKM centered
2 1 0 -1 -2
WT
KO
I
H3K27ac-ChIP-seq
GATA6 +MEIS
MEIS
GATA6
LogRPKM centered
0.6 0.4 0.2 0 -0.2 -0.4 -0.6
WT
KO
J
GATA4 ChIP-seq
GATA e-1288
MEIS e-259
TEAD e-196
MEF2D e-166
NKX2-3 e-112

© The Author(s)

Figure EV4. (**A**) Top motifs, ranked by significance, identified in differential ATAC WT > KO (regions with lower accessibility in MEIS KO) using de novo motif discovery (Homer). (**B**) Top motifs, ranked by significance, identified in regions that increase accessibility in cardiac progenitors relative to mesoderm; data sourced from (Bertero et al, 2019b) using de novo motif discovery (Homer). (**C**) Differentially expressed TFs in MEIS KO. Log FC WT versus MEIS KO at d5 and d7. (**D**) Normalized RNA-seq counts for GATA family members in d5 cardiac progenitors. (**E**) Intersection of GATA6 binding in d5 WT and MEIS KO progenitors. The Venn diagram was generated using all statistically significant GATA6 peaks identified using Motif2site (Zarrineh et al, 2022). (**F**) UCSC tracks of MEIS1 (green) and GATA6 in wild-type and MEIS KO cells (red and black, respectively) at the *NRP1* and *HOXB1* loci. (**G**) Non-proportional Venn diagram generated using MEIS1 and GATA6 top peaks at d5 (FE > 10). High-confidence MEIS1-GATA6 co-occupied regions are linked to genes whose loss of function in mouse leads to heart looping failure. (**H**) ATAC-seq average logRPKM values across replicates, mean-centered across WT and MEIS KO samples. The total number of regions is 142411, grouped into GATA6 + MEIS1 (16315), MEIS1 only (9747) and GATA6 only (27242). (**I**) H3K27ac ChIP-seq average logRPKM values across replicates, mean-centered across WT and MEIS KO. Short reads were counted in open chromatin regions from ATAC-seq; the number of regions is as in (**H**). (**J**) Top motifs, ranked by significance, enriched in GATA4 ChIP-seq in d6 iPSCs-derived cardiac progenitors; data sourced from (Gonzalez-Teran et al, 2022b) using de novo motif discovery (Homer).

© The Author(s)

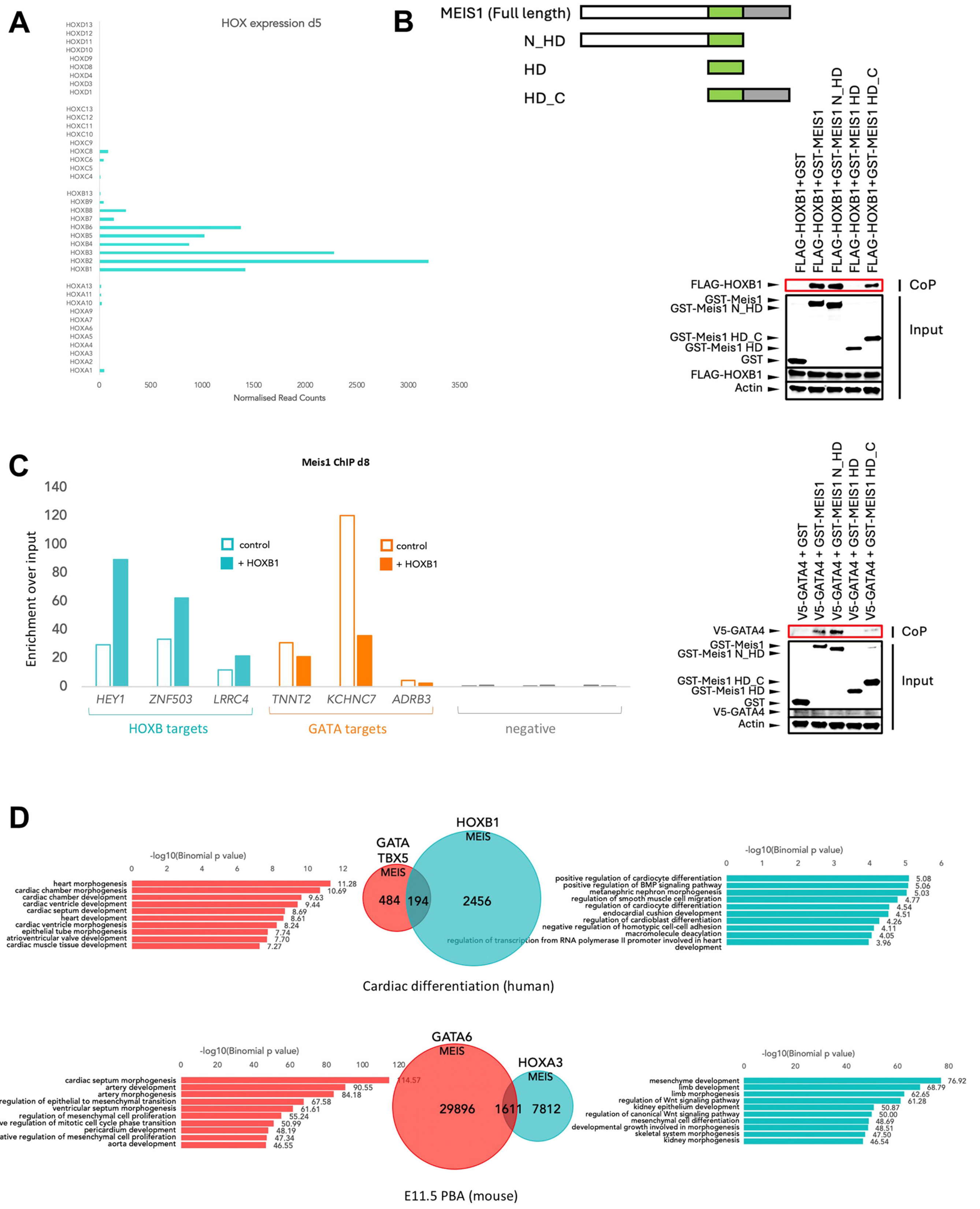
A
HOX expression d5
Normalised Read Counts
B
MEIS1 (Full length)
N_HD
HD
HD_C
FLAG-HOXB1+GST
FLAG-HOXB1+GST-MEIS1
FLAG-HOXB1+GST-MEIS1 N_HD
FLAG-HOXB1+GST-MEIS1 HD
FLAG-HOXB1+GST-MEIS1 HD_C
FLAG-HOXB1
GST-Meis1
GST-Meis1 N_HD
GST-Meis1 HD_C
GST-Meis1 HD
GST
Actin
CoP
Input
C
Meis1 ChIP d8
Enrichment over input
control
+ HOXB1
HEY1 ZNF503 LRRC4 TNNT2 KCHNC7 ADRB3
HOXB targets
GATA targets
negative
V5-GATA4 + GST
V5-GATA4 + GST-MEIS1
V5-GATA4 + GST-MEIS1 N_HD
V5-GATA4 + GST-MEIS1 HD
V5-GATA4 + GST-MEIS1 HD_C
V5-GATA4
D
-log10(Binomial p value)
GATA TBX5 MEIS
HOXB1 MEIS
484 194 2456
Cardiac differentiation (human)
GATA6 MEIS
HOXA3 MEIS
29896 1611 7812
E11.5 PBA (mouse)

Figure EV5. (**A**) Normalized RNA-seq counts for HOX family members in d5 cardiac progenitors. (**B**) Co-precipitation assays. HEK293 cells were co-transfected with expression vectors for GST alone or the following GST-tagged MEIS1 deletions: MEIS1 N_HD (including the N-terminal and HD, boxed in black), MEIS1 HD (HD only, green) and MEIS1 C_HD (including the C-terminal and HD, dark gray) and FLAG-tagged HOXB1 (top) or V5-tagged GATA4 (bottom). Protein interactions were assayed by co-precipitation on glutathione beads directed toward the GST tag and eluted proteins analyzed by western blotting to detect the presence of FLAG-HOXB1 and V5-GATA4 (red box, CoP). No binding was detected with the HD alone, possibly due to incorrect folding. Cell lysates were analyzed by western blotting prior to co-precipitation to detect protein expression (input), including ubiquitously expressed actin, used as a control. (**C**) One of the experiments shown in Fig. 5G, where MEIS enrichment over input is measured at HOXB1 and GATA target enhancers in d8 control and HOXB1-overexpressing cells. Negative control loci are regions with no detectable MEIS1-binding signal, located in the vicinity of *HOXC11*, *AMO3* and *SMAD6*. (**D**) Overlap between MEIS-HOX and MEIS-GATA co-occupied regions. To assess the full extent of the overlap, MEIS-HOX and MEIS-GATA co-bound regions were detected on a genome-wide scale using Motif2Site (Zarrineh et al, 2022). The set of GATA regions included those co-occupied by GATA6 (this paper) and GATA4-TBX5 regions (Gonzalez-Teran et al, 2022b) in cardiac progenitors. GATA-MEIS-only regions are specifically associated with cardiac differentiation GO terms, while HOX-MEIS only regions are associated with regulatory GO terms, which include regulation of cardiac differentiation as well as regulation of other processes. (**D**) Overlap of GATA6, MEIS and HOXA3 binding in the cardiac neural crest-populated posterior branchial arches (PBA) (Losa et al, 2017b). Similar to human cardiac differentiation, GATA6-MEIS binding is highly cardiac-specific while MEIS-HOX is linked with diverse developmental processes. The length of the bars corresponds to the binomial raw (uncorrected) *P* values calculated by GREAT using a Binomial Test.

© The Author(s)
